# Multispecies for multifunctions: combining four complementary species enhances multifunctionality of sown grassland

**DOI:** 10.1038/s41598-021-82162-y

**Published:** 2021-02-15

**Authors:** Matthias Suter, Olivier Huguenin-Elie, Andreas Lüscher

**Affiliations:** Agroscope, Forage Production and Grassland Systems, Reckenholzstrasse 191, 8046 Zurich, Switzerland

**Keywords:** Agroecology, Grassland ecology, Ecosystem services

## Abstract

Assessing the overall performance of ecosystems requires a quantitative evaluation of multifunctionality. We investigated plant species diversity effects on individual functions and overall multifunctionality in a grassland experiment with sown monocultures and mixtures comprising four key grass and legume species. Nitrogen fertilisation rates were 50, 150, and 450 kg N ha^−1^ yr^−1^ (N50, N150, N450). Ten functions were measured representing forage production, N cycling, and forage quality, all being related to either productivity or environmental footprint. Multifunctionality was analysed by a novel approach using the mean log response ratio across functions. Over three experimental years, mixture effects benefited all forage production and N cycling functions, while sustaining high forage quality. Thus, mixture effects did not provoke any trade-off among the analysed functions. High N fertilisation rates generally diminished mixture benefits. Multifunctionality of four-species mixtures was considerably enhanced, and mixture overall performance was up to 1.9 (N50), 1.8 (N150), and 1.6 times (N450) higher than in averaged monocultures. Multifunctionality of four-species mixtures at N50 was at least as high as in grass monocultures at N450. Sown grass–legume mixtures combining few complementary species at low to moderate N fertilisation sustain high multifunctionality and are a ‘ready-to-use’ option for the sustainable intensification of agriculture.

## Introduction

Ecosystem multifunctionality has recently become a major research topic through the need to evaluate the overall performance of ecosystems regarding their processes and the provision of functions and services^1,2^. In agroecosystems, there is evidence that higher plant species diversity promotes many relevant key ecosystem functions, such as nutrient provision and biomass production^3–5^. Because many functions can benefit from an increase in plant species number, higher species diversity is also thought to enhance overall multifunctionality^6–9^, the effect arising primarily because species that are redundant for one ecosystem function under given environmental conditions can play a distinct role for another function or under different conditions. Moreover, species interact with each other to affect multiple functions, which can modify the degree of multifunctionality^10^. The concept of multifunctionality has been widely applied, however to date, there is no single accepted definition of multifunctionality, nor a general agreement on how to measure it^1,2^. Here, we use ‘function’ and ‘multifunctionality’ in the broad sense to refer to ecosystem processes and services, including fluxes of energy and matter.

Previous studies have related species diversity either to an overall multifunctionality index (e.g. Maestre et al*.*^11^, Lefcheck et al*.*^8^) or to a list of several individual functions (e.g. Allan et al*.*^3^). There are, however, situations where evaluating overall multifunctionality and assessment of individual functions, all in a statistically rigorous manner, would be important and beneficial. For example in agronomic systems, which can be designed and generally comprise one or only few plant species selected for particular purposes, knowledge of the species’ single functional performances along with their influence on overall multifunctionality are essential to improve decisions regarding mixture design and production. An integrated assessment is even more important because overall and single functional performances can have diverging responses to changes in the environment^12^, the effect being caused by trade-offs among species contributions to different functions that do not enhance overall multifunctionality^10^. Diverging functional performances can, however, be unravelled by the multivariate modelling framework, which allows for simultaneous testing of species and environmental effects on many functions while taking into account the correlations among functions^13^. It has been acknowledged that the multivariate modelling framework has several advantages over other statistical approaches^2^ and, in particular, avoids problems arising from averaging functions^12,14^. However, this method does not include a measure of overall multifunctionality. Thus to date, a framework for integrated testing of overall multifunctionality and individual functions in their response to abiotic and biotic factors is lacking.

Intensively managed, nutrient rich grasslands have high economic importance for ruminant production^15,16^ and are essential for providing the forage quality needed for ruminant breeds of high genetic merit. Such grasslands naturally contain fewer plant species than nutrient poor, extensively managed grasslands because only a limited number of species can cope with the short defoliation intervals in intensively managed systems^17^. Also, applied fertilisers can contribute to lower species richness through competitive exclusion^18^. From a societal perspective, ecosystem services differ greatly between extensive and intensive systems: while nature conservation, touristic and recreational values are more relevant in extensive systems, production at maximal efficiency has the greatest importance in intensively managed systems, i.e. they should maximise food and feed output while keeping usage and losses of energy and nutrients as low as possible. Whether the diversity-multifunctionality relationship observed in extensively managed systems^8,9^ also holds for more productive grasslands has yet to be demonstrated, given the different services in focus and the comparably lower number of highly specialised species that occur in production-oriented grasslands.

Inorganic nitrogen (N) fertilisers are commonly applied to sown grasslands consisting of high-yielding grass cultivars, with the aim of increasing forage yield. However, the provision of inorganic N fertilisers comes with high environmental costs, as their production needs large amounts of energy^19^ and their application can result in substantial N losses as nitrate^20^ and nitrous oxide to the environment^21,22^. Moreover, N efficiency (N output relative to N input) of such systems should be questioned^23–25^. Nitrogen losses could be reduced and N efficiency enhanced by cultivating mixtures of grasses and legumes in place of highly N fertilised pure grass stands. Grass–legume systems have been studied for single functions (reviewed in Lüscher et al*.*^26^, Phelan et al*.*^27^) and have been shown to increase biomass yield and yield stability^28,29^, increase weed suppression^30–32^, and enhance total N yield^33^ as compared to the average of the monocultures of all plant species used in the mixtures. All of these results imply a potential of plant diversity to substitute for N fertiliser application to grasslands (discussed in Weisser et al*.*^34^ for low-input systems). However, the diversity-multifunctionality relationship has never been evaluated in highly productive grass–legume mixtures, nor has the effect of N fertilisers on multifunctionality been tested. Demonstrating increased multifunctionality in grass–legume mixtures through increased forage yields of high quality, at increased N efficiency and reduced environmental impacts would encourage selection of these systems as an option for sustainable intensification^35,36^.

Here, we investigate effects of species diversity, sown species proportions, and different rates of N fertiliser application on multifunctionality in an intensively managed grassland. We analysed data from a diversity experiment that was established with sown monocultures and mixtures of two grass and two legume species at three levels of N fertilisation (50, 150, 450 kg N ha^−1^ yr^−1^; N50, N150, N450, respectively; see Supplementary Table S1), and was maintained for 3 years^37^. The species were chosen for their expected complementarity with respect to N acquisition and development pattern over years. Ten key functions were measured representing forage production, N cycling, and forage quality; all functions being related to either productivity or environmental impact (Table 1). The multivariate modelling framework was applied to simultaneously test for species interactive effects on multiple functions and the relative importance of multifunctionality drivers^13^. Because this method does not contain a measure of overall multifunctionality, we developed a framework to test overall multifunctionality using a mean log response ratio (e.g., of higher diversity mixtures against monocultures), and we demonstrate that this measure successfully complements the many advantages of the multivariate framework. Given an intensively managed grassland for forage production, we primarily wished to know whether increasing species diversity from monocultures to mixtures with four complementarity species enhances overall multifunctionality. Specifically, we investigated (i) whether species complementarity effects on individual functions result in beneficial mixture effects driving the overall diversity-multifunctionality relationship, (ii) how sown species’ proportions affect overall multifunctionality, and (iii) whether increased N fertilisation weakens beneficial mixture effects on individual functions and overall multifunctionality.

**Table 1:** Overview of functions measured in the experiment.

Function	Abbreviation	Unit	Remarks
**Forage production**			
Aboveground biomass yield	Yield	t ha^−1^ yr^−1^	Dry matter (DM)
Standard deviation of yield	SD_yield_	t ha^−1^ yr^−1^	(i) Across years: year-to-year SD; (ii) Single years: seasonal SD
Temporal stability of yield	Stability	ratio	(i) Across years: averaged annuals yields divided by year-to-year SD (ii) Single years: annual yield divided by seasonal seasonal SD
Aboveground biomass of weeds	Weed biomass	t ha^−1^ yr^−1^	
**N cycling**			
Symbiotic N_2_ fixation	N_sym_	kg ha^−1^ yr^−1^	In harvested biomass
N efficiency	–	ratio	N yield divided by N applied
NO_3_ in soil solution	NO_3_	mg liter^−1^	Expressed in mg N liter^−1^; years 2 and 3 only
**Forage quality**			
Crude protein content	CP	g kg^−1^ DM	
Organic matter digestibility	OM digestibility	g kg^−1^ DM	Years 2 and 3 only
Metabolisable energy content	ME	MJ kg^−1^ DM	Years 2 and 3 only

## Results

### Interactions between species led to beneficial mixture effects on individual functions

**Figure 1:**
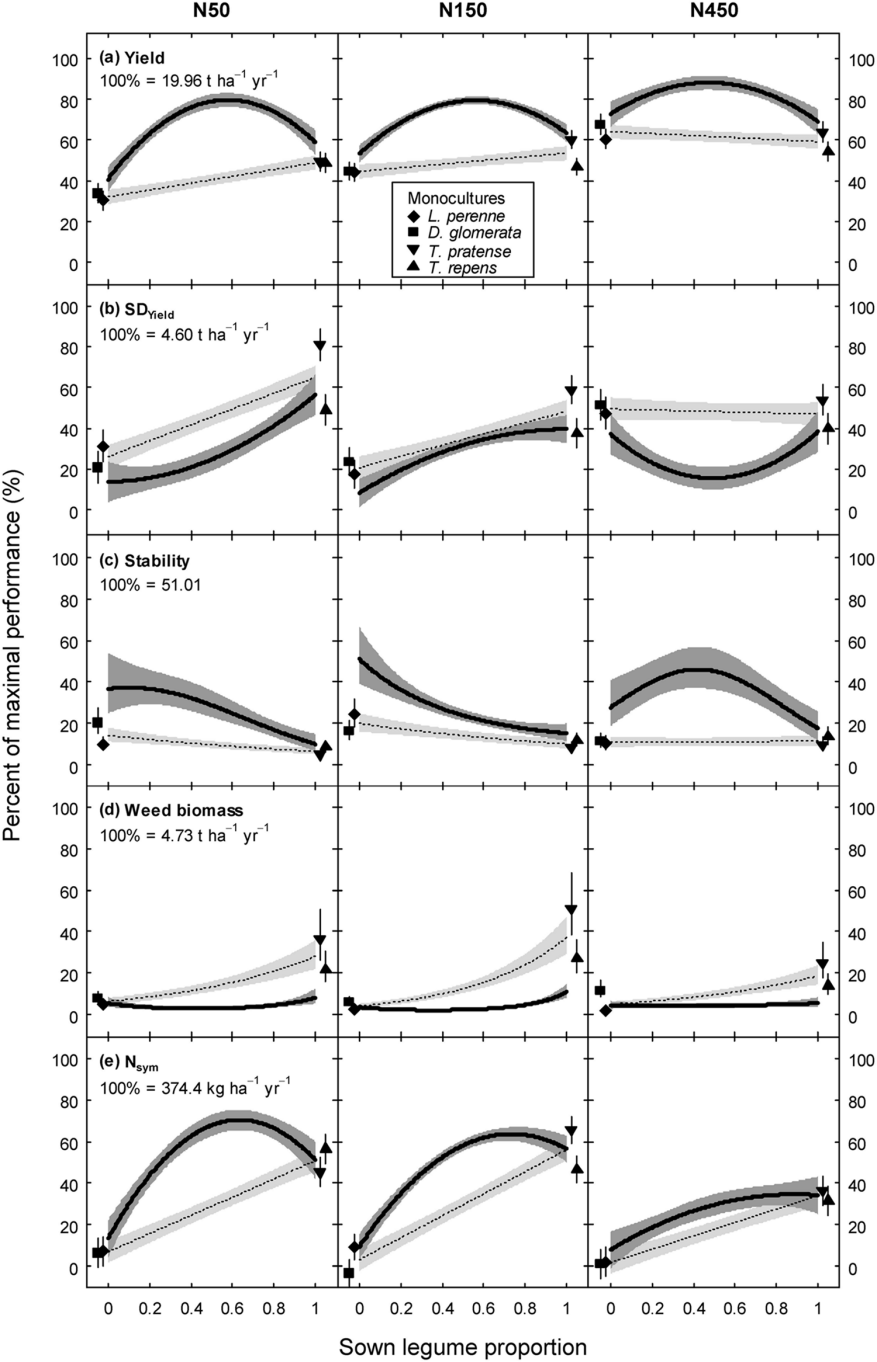

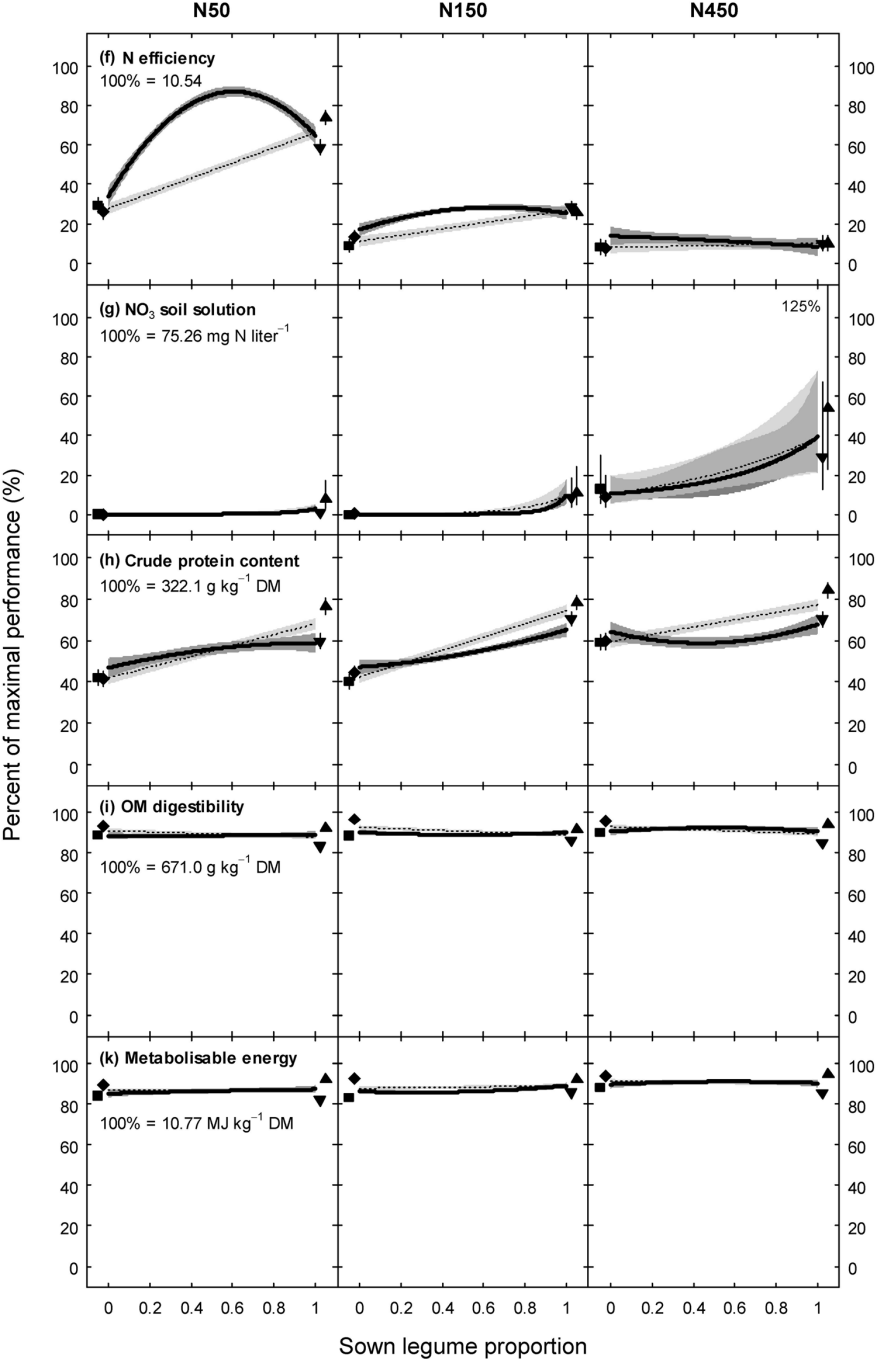
Predicted performance (bold lines, ± 1 s.e. dark grey shaded) of ten functions (**a**–**k**) in dependence on legume proportion at three N fertilisation treatments, data averaged across years (scaled in % of the maximal performance per function at a single year over the three-year experiment and N fertilisation treatments; N50: 50 kg N ha^−1^ yr^−1^, N150: 150 kg N ha^−1^ yr^−1^, N450: 450 kg N ha^−1^ yr^−1^). Predictions and s.e. are based on multivariate regression analysis (Table 2); the underlying model (Eq. 3) explained 88% of the variation in the data (see Supplementary Table S2). Lines display mixtures that are comprised of equal proportions of the two grass and the two legume species, meaning that predictions at a legume proportion of 0.5 are for the four-species equi-proportional mixture and at the left and right endpoints of lines for binary mixtures. Predicted performance of monocultures (± 1 s.e.) is indicated by symbols. Dotted lines display the functional performance (± 1 s.e. light grey shaded) that can be expected from the weighted average of monocultures in the absence of any diversity effect. Stability, weed biomass, and NO_3_ in soil solution are back-transformed to linear scale. The large upper s.e. for the monoculture in panel g) is truncated and reported as a number, and the intermediate gray in g) indicates the cross-section of the two s.e. bands. See Supplementary Fig. S6 for observed versus predicted values.

Over the 3 years, we observed strong diversity effects for individual functions in the mixtures as compared to the average of monocultures. Specifically, diversity effects were the result of beneficial interactions between grass and legume species for the majority of forage production and N cycling functions for all N fertilisation treatments, particularly at N50 and N150 (D_BGL_ effect, Table 2, functions yield, SD_yield_, stability, weed biomass, N_sym_, N efficiency, and NO_3_). Generally, the D_BGL_ effect was larger than pairwise interactions between the fast-establishing and temporally persistent species (Lp∙Dg and Tp∙Tr effects) (Table 2). This result warranted display of functional performances along a range of legume proportions (Fig. 1) to highlight interactive effects between grasses and legumes. Nevertheless, there were also beneficial interactions between fast-establishing and temporally persistent species, namely on yield (Fig. 1a, Table 2: Lp∙Dg and Tp∙Tr effect), stability (Fig. 1c, Table 2: Lp∙Dg effect), and weed biomass (Fig. 1d, Table 2: Tp∙Tr effect), all of which are highly important for sustainable production. This resulted in considerable diversity effects also in the binary grass-grass and legume-legume mixtures.

**Table 2:** Predicted performance of ten functions in monocultures of four forage species and the four-species equi-proportional mixture at three N fertilisation treatments (N50: 50 kg N ha^−1^ yr^−1^, N150: 150 kg N ha^−1^ yr^−1^, N450: 450 kg N ha^−1^ yr^−1^). Data averaged across years. Values are in % of the maximal performance per function (max) at a single year over the three-year experiment and N fertilisation treatments.

Function	Maximal performance per function (max)	N treatment	Monocultures^‡^					Equi-proportional mixture			
			Performance (% of max)					Performance	Diversity effects^§^ (% of max)		
			Lp	Dg	Tp	Tr	Average	(% of max)	D_BGL_ effect	Lp∙Dg effect	Tp∙Tr effect
Yield	19.96 (t ha^−1^ yr^−1^)	N50	30.4	33.9	49.3	48.6	40.6	79.0	33.8***	2.1^†^	2.5^†^
		N150	44.3	44.9	60.2	47.0	49.1	79.2	25.5***	2.1^†^	2.5^†^
		N450	60.5	67.8	64.0	54.5	61.7	88.1	21.8***	2.1^†^	2.5^†^
		s.e.^¶^	4.47	4.47	4.47	4.47	2.52	2.63	4.02	1.31	1.31
SD_Yield_	4.60 (t ha^−1^ yr^−1^)	N50	31.3	20.8	81.0	48.9	45.5	24.7	− 15.5^†^	− 3.1^ns^	− 2.2^ns^
		N150	17.4	23.7	58.8	37.6	34.4	31.7	2.6^ns^	− 3.1^ns^	− 2.2^ns^
		N450	47.4	51.4	54.0	40.0	48.2	15.5	− 27.4***	− 3.1^ns^	− 2.2^ns^
		s.e	7.27	7.27	7.27	7.27	4.10	4.28	6.53	2.13	2.13
Stability	–^#^	N50	41.6	59.7	24.5	37.7	40.9	68.4	18.9*	6.0**	2.7^ns^
		N150	64.1	53.9	37.4	45.9	50.3	63.6	4.6^ns^	6.0**	2.7^ns^
		N450	42.1	45.1	40.9	49.3	44.3	79.8	26.8**	6.0**	2.7^ns^
		s.e	7.22	7.22	7.22	7.22	4.07	4.26	6.53	2.11	2.11
Weed biomass	–^#^	N50	40.9	51.6	87.9	75.7	64.0	28.4	− 27.5**	− 0.8^ns^	− 7.3***
		N150	25.9	46.4	96.1	80.9	62.3	23.0	− 31.2***	− 0.8^ns^	− 7.3***
		N450	19.7	61.1	79.0	65.1	56.2	36.8	− 11.3^ns^	− 0.8^ns^	− 7.3***
		s.e	7.39	7.39	7.39	7.39	4.18	4.37	6.71	2.15	2.15
N_sym_	374.4 (t ha^−1^ yr^−1^)	N50	7.4	6.5	45.3	56.5	28.9	67.8	37.3***	1.6^ns^	0.1^ns^
		N150	9.2	< 0.1	65.6	46.7	29.6	58.2	26.9***	1.6^ns^	0.1^ns^
		N450	2.2	1.2	36.3	31.6	17.8	29.6	10.2^ns^	1.6^ns^	0.1^ns^
		s.e	6.62	6.62	6.62	6.62	3.75	3.91	6.00	1.90	1.90
N efficiency	10.54	N50	26.1	29.2	58.7	73.9	46.9	85.3	37.3***	1.5^ns^	− 0.4^ns^
		N150	13.3	8.7	28.1	25.6	18.9	27.7	7.7***	1.5^ns^	− 0.4^ns^
		N450	7.5	8.3	9.9	10.0	8.9	11.4	1.5^ns^	1.5^ns^	− 0.4^ns^
		s.e	3.46	3.46	3.46	3.46	1.96	2.05	3.17	1.00	1.00
NO_3_ soil solution	–^#^	N50	13.4	21.6	52.6	75.1	40.7	29.4	− 11.3^ns^	–^‡‡^	–
		N150	34.9	8.0	76.6	79.5	49.8	26.9	− 22.9**	–	–
		N450	76.6	81.2	90.3	97.3	86.3	84.5	− 1.8^ns^	–	–
		s.e	9.09	9.09	9.09	9.09	5.13	5.99	8.93	–	–
Crude protein content	322.1 (g kg^−1^ DM)	N50	41.4	42.2	59.6	76.4	54.9	55.7	2.0^ns^	1.2^ns^	− 2.3*
		N150	44.2	40.2	70.6	78.2	58.3	53.4	− 3.8^†^	1.2^ns^	− 2.3*
		N450	59.5	59.1	70.2	84.0	68.2	58.8	− 8.3*	1.2^ns^	− 2.3*
		s.e	3.56	3.56	3.56	3.56	2.02	2.10	3.22	1.01	1.01
OM digestibility	671.0 (g kg^−1^ DM)	N50	93.3	88.9	83.5	92.2	89.5	88.6	− 0.6^ns^	− 0.6^†^	0.3^ns^
		N150	96.8	88.7	86.2	91.6	90.8	89.0	− 1.5^†^	− 0.6^†^	0.3^ns^
		N450	95.7	90.4	85.0	93.9	91.3	92.5	1.6^ns^	− 0.6^†^	0.3^ns^
		s.e	1.22	1.22	1.22	1.22	0.69	0.72	1.09	0.36	0.36
Metabolisable energy	10.77 (MJ kg^−1^ DM)	N50	89.4	84.3	82.2	92.3	87.1	86.6	− 0.1^ns^	− 0.4^ns^	< 0.1^ns^
		N150	92.5	83.3	86.0	92.2	88.5	86.2	− 1.9*	− 0.4^ns^	< 0.1^ns^
		N450	94.2	88.5	85.5	95.0	90.8	91.2	0.7^ns^	− 0.4^ns^	< 0.1^ns^
		s.e	1.39	1.39	1.39	1.39	0.78	0.82	1.25	0.40	0.40

***P ≤ 0.001, ** *P* ≤ 0.01, * *P* ≤ 0.05, ^†^ *P* ≤ 0.1, ns: not significant.

^‡^Lp: *L. perenne*, Dg: *D. glomerata*, Tp: *T. pratense*, Tr: *T. repens.*

^§^The diversity effect is calculated as the difference between the performance of the equi-proportional four-species mixture and the average of monocultures and is split into the effects of mixing grass and legume species (D_BGL_ effect), mixing the two grasses Lp and Dg (Lp∙Dg effect), and mixing the two legumes Tp and Tr (Tp∙Tr effect).

^¶^Standard errors (s.e.) were calculated from the weighted average of variances at each N fertilisation level.

^#^Stability, weed biomass, and NO_3_ in soil solution were natural log transformed and subsequently scaled to range between 0 and 100%; thus, scaling with the maximal functional performance is not reasonably possible. See Fig. 1 for back-transformed values on the linear scale.

^‡‡^Lp∙Dg and Tp∙Tr effects were omitted for NO_3_ in soil solution due to singularity in the design matrix.

**Figure 2:**
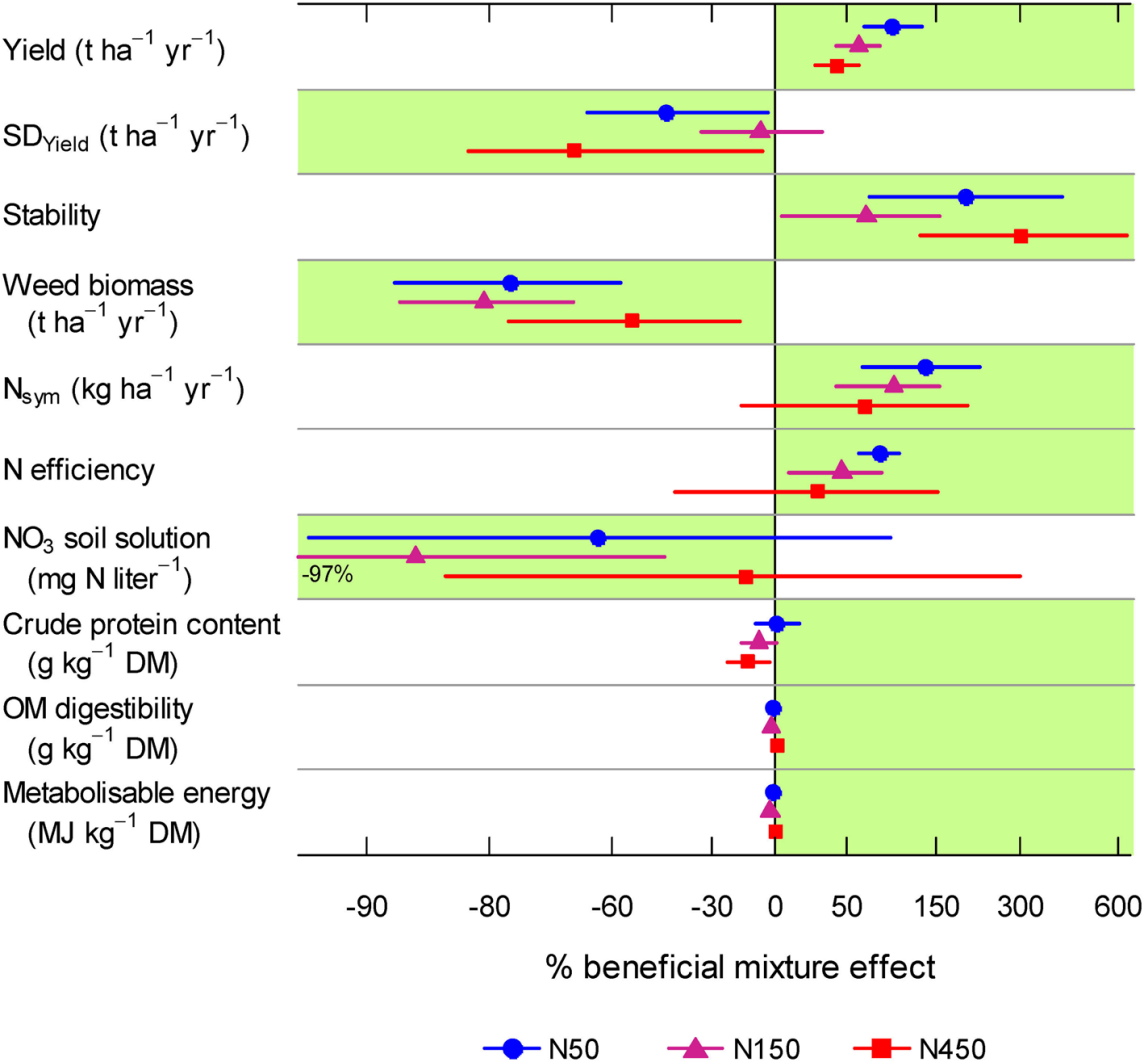
Percent of beneficial mixture effect (mixture performance greater than the average of monocultures) of the four-species equi-proportional mixture for ten functions at three N fertilisation treatments (N50: 50 kg N ha^−1^ yr^−1^, N150: 150 kg N ha^−1^ yr^−1^, N450: 450 kg N ha^−1^ yr^−1^). Data were averaged across years. The preferred direction of the mixture effect is shaded in green. Point estimates are based on multivariate regression analysis (Table 2) and error bars represent 95% confidence intervals (CIs). Functions whose CI does not include 0 can be considered to reveal a significant mixture effect. Stability, weed biomass, and NO_3_ in soil solution were back-transformed to linear scale to calculate beneficial mixture effects. One extreme CI is cut and given as number, and the X axis is log-scaled to equalise distances on both sides of parity.

For all forage production and N cycling functions, the cumulative effect of all species interactions led to beneficial mixture effects at N50 and N150, where mixtures performed better than the weighted average of monocultures over a wide range of legume proportions (evaluated by non-overlapping standard errors of fitted lines, Fig. 1). For example, the four-species equi-proportional reference mixture at N150 exhibited 61% more yield, 8% less variation (SD_yield_), 68% higher stability, 81% less weed biomass, 96% more N_sym_, 46% higher N efficiency, and 87% less NO_3_ compared to averaged monocultures (Fig. 2). Based on the confidence intervals, beneficial mixture effects for forage production and N cycling functions were significant in all but one case at N50 and N150 (each at least *P* ≤ 0.05; Fig. 2; exceptions NO_3_ at N50 and SD_yield_ at N150).

For the three forage quality functions, there were only marginal species interactions (Table 2, Fig. 1h–k) and next to no overall mixture benefits in all N treatments, if functions were expressed as content (g kg^−1^ yield in CP and OM digestibility, MJ kg^−1^ yield in ME, Fig. 2). Notably, this result appeared despite substantially higher yield in mixtures (Fig. 1a). As a result, CP, digestible OM, and ME per hectare (ha^−1^ yr^−1^) were much greater in mixtures than in averaged monocultures, with beneficial mixture effects being in the ranges of 82–122% (N50), 52–72% (N150), and 28–44% (N450) for the four-species reference mixture (Supplementary Fig. S1).

### N fertilisation effects on individual functions

All forage production functions, two of the three N cycling functions (N_sym_ and NO_3_), and the three forage quality functions each performed similarly at N50 and N150 (Fig. 1). However, N efficiency considerably decreased with increasing N fertilisation. In the four-species reference mixture, N efficiency at N50 (= 9.0) was about 3 times greater than at N150 (= 2.9; Fig. 1f) and was about 8 times greater than at N450 (= 1.2). Moreover, N efficiency in the reference mixture was about 3 times (N50) and 2.5 times (N150) greater than in grass monocultures. Regarding NO_3_ in the soil solution, it was negligibly low at both N50 and N150 (< 0.1 mg N liter^−1^), but had distinctly higher concentrations at N450, particularly in legume-dominated mixtures and pure legume stands (Fig. 1g).

### N fertilisation reduced beneficial mixture effects

Generally, increased N fertilisation reduced beneficial mixture effects (Fig. 2), indicated by a highly significant interaction between the N fertilisation treatment and the D_BGL_ effect (Chi square = 132.2, *P* < 0.0001). For example, the beneficial effect on yield was + 95% at N50, but was only + 43% at N450, and corresponding values for N_sym_ were + 135% (N50) and + 66% (N450). The only exceptions were SD_yield_ and stability, for which the mixture benefits were greatest at N450 (Fig. 2).

### Individual functions performance, beneficial mixture effects, and legume proportions at single years

Each of the functions performed fairly similar at each of the three experimental years (Supplementary Tables S3–S5, Figs. S2–S4). Thus, beneficial mixture effects were also evident at each individual year, while increased N fertilisation generally reduced mixture benefits (Supplementary Fig. S5). In conclusion, mixture benefits persisted over the entire experimental period of 3 years and at all three N fertilisation treatments.

At N50 and N150, legume proportions remained fairly constant around 45% for 2 years and decreased to 24% (N50) and 12% (N150) only in the third year, accompanied by an increase in the proportion of grass species. At N450, legume proportions strongly decreased from 32% in the first year to 5% in the third year (see Supplementary Appendix S1 for details).

### Significantly enhanced multifunctionality in mixtures over a wide range of legume proportions

**Figure 3:**
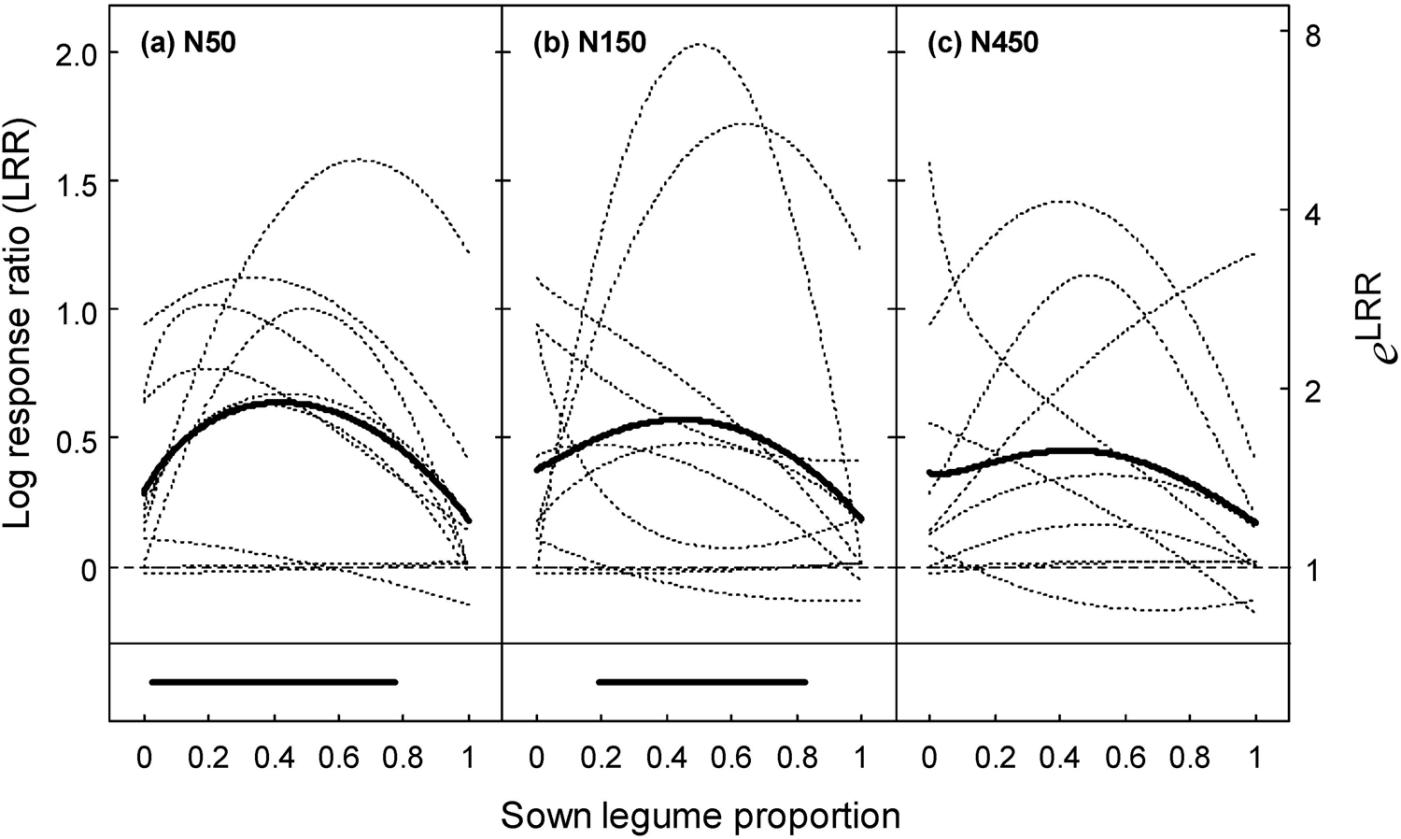
Multifunctionality in mixtures compared to monocultures expressed as the mean log response ratio (MLRR) (bold continuous line) across ten functions (thin dotted lines) at three N fertilisation treatments (**a**) N50: 50 kg N ha^−1^ yr^−1^, (**b**) N150: 150 kg N ha^−1^ yr^−1^, and (**c**) N450: 450 kg N ha^−1^ yr^−1^. Data were averaged across years. The LRR of mixtures *versus* averaged monocultures was calculated for each function with values based on multivariate regression analysis (Table 2) for legume proportions between zero and one, with equal proportions of the two grass and the two legume species. It follows that the LRR at a legume proportion of 0.5 is displayed for the four-species equi-proportional mixture and the LRR at the left and right endpoints of lines is for binary mixtures. The horizontal bold line at the bottom of the panels indicates the range of legume proportion for which the MLRR was significantly different from zero (*P* ≤ 0.05, based on range test^38^).

Overall, beneficial mixture effects were evident without trade-offs between functions (Fig. 2, Supplementary Fig. S1), indicating clear complementarity of species in terms of their contribution to functional performances. As a result, at N50 and N150, we observed enhanced multifunctionality in mixtures over a wide range of legume proportions (Fig. 3a,b, indicated by the MLRR). Multifunctionality was significantly enhanced in mixtures with legume proportions between 0.03 and 0.78 (N50) and 0.19 and 0.83 (N150) (*P* ≤ 0.05, based on range test^38^), with the four-species reference mixture (legume proportion: 0.5) having on average 1.9 times (N50) and 1.8 times (N150) the performance of averaged monocultures. Notably at N50, binary grass-grass mixtures also enhanced multifunctionality over monocultures by a factor of 1.3 (*t*_9_ = 2.07, *P* = 0.069). At the very high N level of N450, the MLRR was generally smaller (Fig. 3c), but the four-species reference mixture had still 1.6 times the performance of monocultures (*t*_9_ = 2.22, *P* = 0.053). Taken together, the results indicated consistent benefits of mixtures on multifunctionality over monocultures for greatly varying legume proportions at low to moderate N fertilisation.

### Higher multifunctionality in lowly fertilised mixtures than in highly fertilised grass monocultures

**Figure 4:**
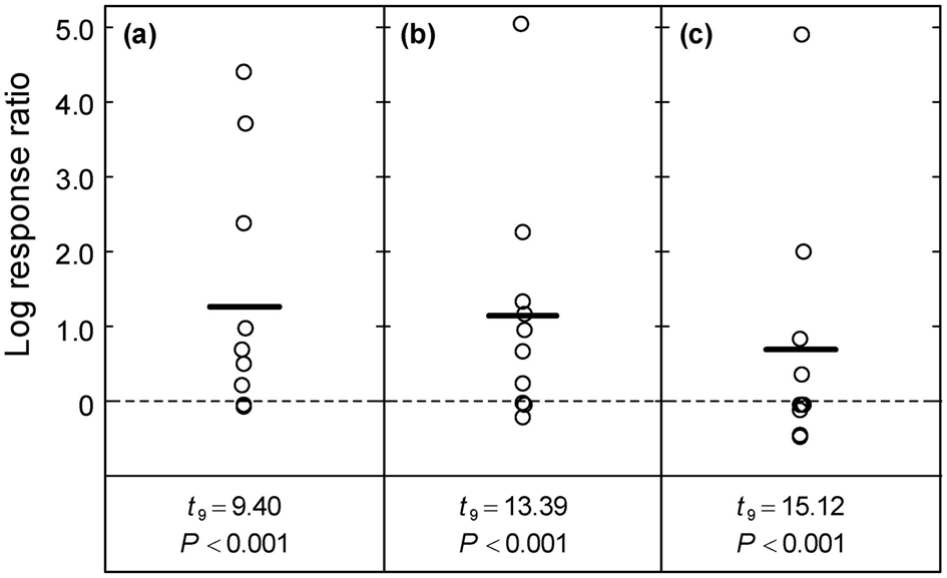
Multifunctionality in mixtures at N50 compared to different types of communities at N450 expressed as the mean log response ratio (MLRR) (horizontal line) across ten functions (circles). The log response ratio for each function was calculated for the four-species equi-proportional mixture at N50 against (**a**) the average of the two grass monocultures at N450, (**b**) the average of all monocultures at N450, and c) the four-species equi-proportional mixture at N450 (N50: 50 kg N ha^−1^ yr^−1^, N450: 450 kg N ha^−1^ yr^−1^). Data were averaged across years, and LRRs of functions were calculated based on multivariate regression analysis (Table 2). The inference (*t*- and *P*-values) refers to a test of the MLRR against zero. Circles are scattered horizontally to improve their visibility.

Testing directly the effect of N fertilisation on multifunctionality revealed that the four-species reference mixture at N50 significantly outperformed the average of the two grass monocultures at N450 (Fig. 4a, *t*_9_ = 9.40, *P* < 0.001), and had also higher multifunctionality than the average of all monocultures and the four-species reference mixture at N450 (Fig. 4b,c). Because the very low NO_3_ concentrations in the soil solution of the reference mixture at N50 (Fig. 1g) led to high LRR values (topmost LRR for each comparison, Fig. 4), the tests were repeated without this function. In doing so, the MLRR was still positive in all three cases but became non-significant (*P* > 0.05). This led us to conclude that the degree of multifunctionality of the four-species reference mixture at N50 was at least as high as in (grass) monocultures and mixtures at N450, yet with an N fertiliser application nine times less.

## Discussion

We found multifunctionality to be considerably enhanced in four-species mixtures compared to monocultures of intensively managed grassland. This increase was significant over a wide range of legume proportions at low to moderate levels of N fertiliser application. Most importantly, we observed no trade-offs between individual functions, meaning that species interacted synergistically in favour of all functions. Beneficial mixture effects were robust over all three experimental years. Our new measure, the MLRR across functions, proved to be valuable in evaluating overall multifunctionality, while the individual drivers of multifunctionality could be identified through the multivariate modelling framework^13^.

### Enhanced multifunctionality in mixtures by targeted combination of four species

Previous studies have shown a positive diversity-multifunctionality relationship for ecosystem processes in low productivity systems with up to 60 species (e.g. Hector and Bagchi^6^, Maestre et al*.*^11^, Lefcheck et al*.*^8^). Here, we extend these findings by demonstrating strongly enhanced multifunctionality in mixtures by increasing plant diversity up to only four species, selecting functions that covered key ecosystem services related to forage production, N cycling, and forage quality. Our selected species were cultivars known to perform well in pure stands under intensively managed conditions. However, it is not a priori clear how such species perform in mixtures and how their combination affects multifunctionality, as multifunctionality also depends on the species’ dominance hierarchy and competitive interactions within the community, as well as on the combination of species’ traits. It is thus remarkable that multifunctionality was enhanced by a factor of almost two by using only four species, and we attribute this strong diversity effect to the targeted combination of species with complementary functional traits^39,40^.

The strategy of targeted combination of few species as done in our experiment clearly differs from the random species assemblage design of many biodiversity experiments in low productivity systems, which often contain more than 15 species (e.g. Zavaleta et al*.*^41^, Weisser et al*.*^34^). Theoretically, each additional species could provide benefits for functions poorly supported by the other species. However, the number of species necessary to optimise overall multifunctionality becomes increasingly uncertain with an increasing number of functions expected from the mixture^10^. Moreover, conditions that maximise a particular function’s performance might decrease the performance of other functions^12^. In sown grasslands, which are characterized by the cultivation of few (or one) species under a management that optimises production, one would preferably add only few species to promote more functions, as benefits and costs should be balanced. It is thus of high practical importance that increasing diversity from one to only four species has already demonstrated a substantial benefit on overall multifunctionality. Future work should investigate whether the inclusion of more species and/or functional groups (e.g., forbs) in intensively managed grasslands further enhances multifunctionality, and how mixture advantages on multifunctionality shape up when more functions are considered, e.g., soil C sequestration, nitrogen denitrification, or soil fauna diversity.

### Drivers of multifunctionality

Our four species were complementary in their manner of N acquisition and temporal establishment, and these differing traits formed the basis for the beneficial interactions between the species (Fig. 1, Table 2). Much of the positive grass–legume interactions can be explained by the process of symbiotic N_2_ fixation of legumes and related benefits to the grass partners. While grass species stimulate symbiotic N_2_ fixation in legumes, the presence of legume species stimulates the uptake of N from non-symbiotic sources in the grass partners and allows for transfer of symbiotically fixed legume N to grasses^33,42,43^. Moreover, symbiotic N_2_ fixation is up-regulated, where N demand is large^44,45^, as in mixtures with a large share of grass and low N fertilisation^33,43^. ‘Regulation of N_2_ fixation by demand’ can thus well explain the increase in multifunctionality over a large range of legume mixture proportions at both N50 and N150, and thus at very differing N demands (Fig. 3a,b). Importantly, benefits of legume presence to biomass yield, N yield, phosphorus yield, and CP content can sustain even past the actual cultivation year through soil-transferred legacy effects^46,47^, which explains our robust mixture benefits to multifunctionality despite decreasing legume proportions in the third production year.

Interactions between the fast-establishing and temporally persistent grass and equally between the two legume species contributed to enhanced multifunctionality in mixtures (Fig. 3a,b, legume proportion 0 and 1). If the species’ differences in temporal development were the sole driver of these interactions, one would expect a larger grass-grass and legume-legume effect across the 3 years than for the single years. This was observed for weed biomass and to a lesser extent for yield (Tp∙Tr effect), but neither for the other functions nor the Lp∙Dg effect (compare Table 2 and Supplementary Tables S3–S5). Thus, these important effects can presumably be also assigned to the species’ differing rooting depths^48,49^. Overall, complementarity effects among species enhanced multifunctionality over a wide range of legume proportions, which is an important outcome for practical grassland management because it demonstrates sustained benefits from mixtures despite fluctuation of legume proportion.

### High rates of N fertilisers diminish mixture benefits on multifunctionality

Nitrogen application rates of N50 and N150 resulted in comparable mixture benefits on multifunctionality; however, the very high rate of N450 clearly attenuated these gains (Fig. 3). Even worse, N450 lead to critically enhanced levels of NO_3_ in the soil solution and thus adverse impacts on the environment (Fig. 1g). Previous studies have revealed a percentage decrease of the diversity effect on yield with high N fertilisation at high and low productive systems^37,50,51^. Yet, the detrimental effects of high N fertilisation on species diversity gains to yield and other ecosystem functions are only partly understood. In our grassland, fertilisation of N450 has induced a shift in community proportions towards the two grass species^37^ (Supplementary Appendix S1), which probably reduced the degree of species complementarity effects. In addition, strongly decreasing symbiotic N_2_ fixation at N450 (Fig. 1e) must have caused a loss of the legumes’ advantage for N provisioning and beneficial grass–legume interactions^44^. In line with this explanation, multifunctionality was not negatively affected by N fertilisation in pure grass–grass mixtures (compare Fig. 3, legume proportion 0). In conclusion, beneficial mixture effects at low rates of N fertilisers were so strong that the degree of multifunctionality of the four-species reference mixture at N50 was at least as high as in highly fertilised grass monocultures at N450 (Fig. 4a), although the majority of functions were essentially production-related. Given that highly N fertilised grass monocultures are still practiced in intensive ruminant production systems^25^, this impressive result demonstrates that high productivity and increased multifunctionality can be achieved in grass–legume mixtures with only a moderate use of N fertiliser, which has important implications for sustainable agriculture.

### No trade-offs in our set of functions

Interestingly, we observed no trade-offs among functions although they could be expected in several cases. First, there were distinct yield benefits in mixtures compared to monocultures, along with increased stability (Fig. 1a,c), despite that the latter could be supposed to remain constant or to decrease with increasing biomass yield. Greater biomass yield is usually associated with greater variance (or SD) of yield^52,53^, and so it is surprising that we found equal or lower SD_yield_ in mixtures than in averaged monocultures, leading to higher stability (Fig. 1b,c). Temporal asynchrony in species’ proportions in mixtures has been demonstrated as a relevant mechanism to reduce SD_yield_ and to increase yield stability^29,54^. In our experiment, the yield advantage by mixing species with a differing temporal development must have been so strong that it overrode the expected increase in yield variance. Indeed at N50 and N150, SD_yield_ was lowest and stability largest in binary mixtures of the fast-establishing and temporally persistent grass species (Fig. 1c). Regarding N fertilisation, the application of 450 kg N ha^−1^ year^−1^ to mixtures enhanced their yield only marginally (by about 10% in the equi-proportional mixture compared to N50, Fig. 1a), but substantially increased stability (by about 55%, Fig. 1c). This can be explained by a strong shift in community composition towards the two grass species at N450 (Nyfeler et al*.*^37^), with the grasses under very high N fertilisation providing constantly high yields over time, a feature that has recently been demonstrated in a similar type of grassland under cool maritime conditions^55^.

Second, despite a substantial N input into the system, there were only negligible amounts of NO_3_ in the soil solution (Fig. 1g: N50, N150). At N50 and N150, total N input through N fertilisation and symbiotic N_2_ fixation in the four-species equi-proportional mixture was about 300 and 370 kg ha^−1^ year^−1^, respectively, nevertheless NO_3_ remained < 0.10 mg N liter^−1^ soil solution (equivalent to < 0.44 mg NO_3_ liter^−1^). We argue that there are several reasons why N losses remain small in equilibrated grass–legume mixtures^39^: (i) where the sink of N for growth is marginal or small, the activity of symbiotic N_2_ fixation is down-regulated, (ii) the process of symbiotic N_2_ fixation takes place within the legume nodules and so N is not freely available in a reactive form, and (iii) the grass species effectively take up N from the soil. Our chosen grasses *L. perenne* and *D. glomerata* were specifically selected for their functional complementarity in N acquisition and are known for strong, competitive soil N uptake^33^.

Third, forage quality was not generally reduced in mixtures (Fig. 1h–k), despite significantly increased biomass yields, in agreement with Bélanger et al*.*^56^ and Schaub et al*.*^57^. Consequently, crude protein, digestible OM, and ME per hectare were greater in mixtures in this experiment (Supplementary Fig. S1). Generally, the nutritional quality of forage plants decreases with plant development and maturity, due to a larger proportion of lignified cellulose accompanied with a decrease in CP and other readily digestible quality parameters. Decline in CP and OM digestibility during crop growth is well documented^58^, and thus higher biomass yields may be associated with smaller nutritional quality. Nevertheless, Jones et al*.*^59^ observed a higher OM digestibility in *Phalaris arundinacea* L. when grown in mixtures with legumes than when grown alone, which could explain why the relationship between biomass yield and nutritive quality differs between mixtures and monocultures.

### From individual functions to overall multifunctionality: methodological aspects

A comprehensive evaluation of the diversity-multifunctionality relationship must be at least three-fold: (i) quantifying the effect of species diversity on multifunctionality, (ii) evaluating how species interact to affect multiple functions, which allows identification of the drivers of multifunctionality, and (iii) accounting for the generally correlated nature of the data. Applying the multivariate modelling framework for the first time after its publication^13^, we have demonstrated that this approach, in conjunction with the mean MLRR as a measure of overall multifunctionality, fulfils all of these demands.

Notably, the LRR is an intuitive, widely used, and easy-to-apply measure, and is particularly useful for estimation of effect sizes because of its desirable statistical properties^60^. We did not apply weights to LRRs of individual functions for the calculation of the MLLR, although this could be done to reflect, e.g., the demands of stakeholders^2^. Moreover, our design and measurements proved to be sufficiently detailed to initially investigate all possible pairwise species interactions affecting individual functions (Eq. 2) and then identify the most relevant drivers for increased multifunctionality. In our framework, opposing behaviours between multifunctionality and individual functions in their response to changing diversity and environment would be detected and appropriate conclusions could be drawn for management decisions. We also emphasise that correlations among functions should not be neglected when testing multifunctionality and individual functions. Correlated functions seriously compromise the validity of the statistical inference of the diversity-multifunctionality relationship, as high correlations may merely represent the same underlying functional process that is repeatedly tested. Finally, the MLRR approach is flexible in that it allows the evaluation of multifunctionality regarding different aspects in a system. Here, we have investigated the diversity-multifunctionality relationship by comparing mixtures with monocultures, and have assessed the resource use-multifunctionality relationship by comparing communities at different rates of N fertiliser application; yet, other comparisons would be possible.

The multivariate modelling framework might have limitations when evaluating large number of functions and communities with many species. Given an experiment with many species, Kirwan et al*.*^61^ have suggested strategies to reduce the number of species identity and species interactive terms and thus the number of (fixed) coefficients to be estimated. However, given large number of functions, the number of correlations between them rapidly increases: for *n* functions, *n* × (*n* − 1)/2 (random) correlation parameters must be estimated. A high number of functions (e.g., > 20) might thus become an issue of computing power and/or induce convergence problems. Strategies to simplify the correlation matrix for such situations should be developed (see Niku et al*.*^62^ for a latent variable approach).

### Conclusions: legume-based multispecies grassland systems—a contribution to sustainable agriculture

Here, we have clearly illustrated that grass–legume mixtures increased yields of high forage quality, increased stability, maintained weed suppression, enhanced N efficiency but kept N leaching at low levels, all of which increased multifunctionality. These features align well with recent demands to produce more with same recourses and concomitantly preserve the environment^35,36^. Lüscher et al*.*^26^ have suggested that legumes can potentially contribute to sustainable intensification through, amongst others, (i) increasing forage production, (ii) providing an ‘energy-neutral’ N input into grassland via symbiotic N_2_ fixation, and (iii) increasing the nutritive value and conversion efficiency of herbage. Equally important, the species of our experiment are used worldwide in production-oriented grassland systems and their cultivation in mixtures provides a ‘ready-to-use’ approach. Given the robustness in gains of total biomass and protein yield across wide environmental gradients^28,63^ and the lack of adverse effects on the environment, legume-based multispecies grassland systems should become a key option for the sustainable intensification of agriculture.

## Methods

We used a dataset from a grassland diversity experiment at Zürich-Reckenholz, Switzerland, in the Atlantic central climatic zone of Europe. The data contain measurements on many functions from 78 plots that comprised monocultures and mixtures sown at a wide range of species relative abundances, set up at three levels of N fertiliser application and maintained for 3 years following establishment, which is a typical time in grassland-crop rotations.

Monocultures and mixtures were sown following a simplex design^64^. Four perennial species, known to be key forage species in ruminant production, were selected based on the factorial combination of their functional traits related to temporal establishment (fast-establishing *vs.* temporally persistent), and N acquisition (non-fixing for grasses, N_2_-fixing for legumes). The species were *Lolium perenne* L. cultivar (cv.) Lacerta (fast-establishing grass), *Dactylis glomerata* L. cv. Accord (temporally persistent grass), *Trifolium pratense* L. cv. Merviot (fast-establishing legume), and *Trifolium repens* L. cv. Milo (temporally persistent legume). The type of stands were: monocultures (100% of one species), binary mixtures (50% of each of two species), an equi-proportional mixture (25% of each of the four species), dominant mixtures (70% of the dominant species, 10% of each of the other three), and co-dominant mixtures (40% of each of two species, 10% of each of the other two; see Supplementary Table S1). All types of stands were sown at two levels of overall sown density, with the high level being the recommended seed weight (100%) under conditions typical of Switzerland, and the low level being 60%.

The experiment was sown in August 2002 on plots of 3 m × 6 m and was maintained from 2003 (year 1) to 2005 (year 3). The plots were fertilised with N fertiliser (as NH_4_NO_3_) at rates following a geometric series: 50, 150, or 450 kg N ha^−1^ yr^−1^ (N50, N150, and N450, respectively), split into five equal applications. In early spring, all plots received phosphorus and potassium in amounts expected to be non-limiting for intensively managed grasslands on fertile soils in Switzerland. At the N150 treatment, all types of monocultures and mixtures were established, whereas the N50 and N450 treatments only included the monocultures, the equi-proportional mixture, and the dominant mixtures. The 78 plots were arranged in a fully randomised design. Consult Nyfeler et al*.*^37^ for full details of the experimental design, establishment, and maintenance.

Ten functions were measured representing (i) forage production: aboveground biomass yield, standard deviation of yield, temporal stability, weed biomass; (ii) N cycling: symbiotic N_2_ fixation, N efficiency, NO_3_ in soil solution; and (iii) forage quality: crude protein content, organic matter digestibility, metabolisable energy content (Table 1). To date, detailed analyses from the experiment have been published on two functions, namely biomass yield^37^ and symbiotic N_2_ fixation^33^.

### Measurement of functions

#### *Aboveground biomass yield and weed biomass*

All plots were harvested five times annually at 5 cm above ground surface. Aboveground biomass yield at each harvest was determined by drying a representative subsample to constant weight (65° C for 48 h), and this data was summed to give total annual biomass yield. Biomass proportions of the four sown and pooled unsown species (weeds) were measured by manually separating samples from permanent sub-plots (0.8 m × 0.3 m), which was done at the first, third, and fifth harvest of each year. These data allowed for calculation of weed biomass per ha and year.

#### *Standard deviation and stability of yield*

Year-to-year standard deviation of yield (SD_yield_) was calculated from the annual yields of the three experimental years, and stability was defined as the ratio of averaged annual yields to year-to-year SD_yield_ (following Lehman and Tilman^65^). To measure yield variation within each year, seasonal SD_yield_ was calculated from the five annual harvests, and seasonal stability was defined as the ratio of total annual yield to seasonal SD_yield_. We purposely use both SD_yield_ and stability as both measures are essential to evaluate yield variation^66^.

#### *SymbioticN*_*2*_*fixation*

Symbiotic N_2_ fixation (N_sym_) was determined by the isotope dilution method^67^. Double-labelled ^15^N-enriched ^15^NH_4_^15^NO_3_ was applied on a permanently defined, central part of each plot (1.4 m × 1.5 m). Plant samples were analysed for ^15^N and ^14^N abundance by gas isotope ratio mass spectrometry and by thermal conductometry. N_sym_ in the sward, as calculated here, comprises legume N derived from the atmosphere (N_dfa_) plus N derived from apparent N_dfa_ transfer to the grass (N_trans_). See Supplementary Appendix S1 and Nyfeler et al*.*^33^ for full details of measurements and calculations.

#### *N efficiency*

N efficiency was defined as the ratio of total N yield to the amount of applied fertiliser N and therefore measures the total N output of the system in relation to the fertiliser N input. Total N yield was calculated by first multiplying N content from biomass samples with their total dry mass to give the N yield per harvest. Annual total N yield was then computed as the sum of all harvests.

#### *NO*_*3*_* in soil solution* (NO_3_)

Porous cup tension lysimeters were installed to extract soil water from a depth of 60 cm below ground surface. In 2-week intervals from October 2004 to April 2006, a suction of 80 kPa was applied 1 day prior to sampling, and concentrations of nitrate–N (NO_3_-N) were determined by spectrophotometry. We note that NO_3_ data were only available for years 2 and 3. See Supplementary Appendix S1 for details of the measurements.

#### *Crude protein content *

Crude protein content (CP) in stand biomass was calculated from the N content in biomass samples, multiplied by 6.25. The justification for the multiplicative factor is given by the fact that all biological proteins contain on average 16% N^68^.

#### *Organic matter digestibility*

Organic matter digestibility (OM digestibility) was determined from biomass samples of the second and fourth harvest following the two-stage in vitro fermentation process with rumen liquor and acidic pepsin solution according to Tilley and Terry^69^; see Supplementary Appendix S1 for details. Information on OM digestibility was only available for years 2 and 3 of the study.

#### *Metabolisable energy content*

Metabolisable energy content (ME) of stand biomass was calculated based on OM digestibility and CP following a reference manual of Agroscope^70^; see Supplementary Appendix S1 for calculation. Due to the connection with the measurement OM digestibility, ME data were only available for years 2 and 3.

Data for each function were computed at the plot level for each of three experimental years (the three exceptions as noted). For analyses across years, data was averaged across available years, except SD_yield_ and stability (see above).

### Data analyses

We applied the multivariate modelling framework^13^ to estimate simultaneously species identity and diversity effects of the ten functions along with effects of N fertilisation. To allow direct comparisons of the model terms, all functions’ data were standardised to a common scale by dividing them by their maximum value (at a single year) over the 3-year experiment and N fertilisation treatments. This scaling allowed for a direct comparison of results among years. Note that the multivariate approach is a generalisation of the univariate diversity interaction model^61^, and we refer to Supplementary Appendix S1 for a summary to the univariate regression.

In the following, we generally refer to the analysis of data averaged across experimental years, and all equations model the response at a single plot (plot subscripts are omitted). A preliminary regression equation was specified for the *k*th function (*k* = 1–10) with:

1 $${y}_{k}={\alpha }_{k}\mathrm{DENS}+\sum_{f=1}^{3}\sum_{i=1}^{4}{\beta }_{ifk}{P}_{i}\times {\mathrm{N}\_\mathrm{Treat}}_{f}+\sum_{\begin{array}{c}i,j=1\\ i<j\end{array}}^{4}{\delta }_{ijk}{P}_{i}{P}_{j}+{\varepsilon }_{k}$$

The *α* coefficient denotes the effect of changing sowing density on the response variable *y*_*k*_, for example biomass yield, with DENS being coded as − 0.5 and 0.5 for the low and high sowing density, respectively, so that all other parameters give the response *y*_*k*_ at average density. Variables *P*_*i*_ denote the species’ sown proportions in a stand. Coefficients *β*_1*fk*_ to *β*_4*fk*_ estimate the effects of the four species’ proportional contributions on *y*_*k*_ (identity effects) and, if *P*_*i*_ = 1, *β* coefficients estimate the response *y*_*k*_ of species’ monocultures. Identity effects *β*_*ifk*_ are estimated at each N fertilisation treatment *f* (factor N_Treat with three levels: N50, N150, and N450), which is equivalent to specifying an interaction between N_Treat and *P*_*i*_. Coefficients *δ*_*ijk*_ estimate the six possible pairwise interactions among the four species to evaluate diversity effects. The residual term *ε*_*k*_ is assumed to be normally distributed with constant variance *σ*^2^_*k*_.

Dooley et al*.*^13^ extended Eq. (1) to a multivariate model using matrix notation, where response variables *y*_*k*_ constitute a matrix of *k* columns. Yet for parameter estimation, the multivariate matrix notation can be re-written applying principles of linear mixed-effects regression^71^, leading to:

2 $${y}_{k}=\sum_{k=1}^{10}{\alpha }_{k}\mathrm{DENS}\times {\mathrm{FUNC}}_{k}+\sum_{k=1}^{10}\sum_{f=1}^{3}\sum _{i=1}^{4}{\beta }_{ifk}{P}_{i}\times {\mathrm{N}\_\mathrm{Treat}}_{f}\times {\mathrm{FUNC}}_{k}+\sum_{k=1}^{10}\sum_{\begin{array}{c}i,j=1\\ i<j\end{array}}^{4}{\delta }_{ijk}{P}_{i}{P}_{j}\times {\mathrm{FUNC}}_{k}+{\lambda \mathrm{Plot}+\varepsilon }_{k}$$

Here, the response variable *y*_*k*_ denotes a column vector, in which performances of all functions *k* are listed. The variable FUNC is a factor with ten levels, one for each function *k*. Consequently, predictor variables DENS, *P*_*i*_, *P*_*i*_*P*_*j*_, and N_Treat (with meanings as explained) are repeated *k* times within columns of the design matrix, and corresponding coefficients are estimated as fixed parameters (see Dooley et al*.*^13^ for an example). The variable ‘Plot’ (also repeated *k* times) estimates the plot-specific, common variance of the functions per plot (random intercept). The term *e*_*k*_ ~ MVN(0, **Σ**), where MVN denotes multivariate normal, with mean 0 and co-variance matrix **Σ** among functions. For parameter estimation, the residual parameter was defined as Var(*e*_*k*_) = *σ*^2^*δ*_*k*_^2^, with *δ* being a ratio to represent *k* variances (see Pinheiro and Bates^71^ p. 209 for details), and an unstructured co-variance matrix was imposed on the residuals to estimate **Σ**.

Applying Eq. (2), it turned out that sowing density had no significant effect (*t* < 1.65, *P* > 0.10 for all functions but one), and it was omitted from all further models. Moreover, to achieve a multivariate normal residual distribution, the functions stability, weed biomass and NO_3_ were first natural log transformed and then divided by their maximum value to range between 0 and 100%. Given this amendment, residuals showed no evidence of a deviation from multivariate normality (approved by Mardia’s multivariate normality test^72^).

Equation 2 estimates six *δ*_*ijk*_ coefficients per function (diversity effects). To increase parsimony, a series of hierarchical models were constructed as described in detail in Nyfeler et al*.*^37^ and Helgadóttir et al*.*^55^. Applying likelihood ratio tests for the comparison of nested models (see Pinheiro and Bates^71^ p. 83), it appeared that the six diversity effects could be grouped together to represent specific interactions between grass and legume species (D_BGL_), and interactions between the two grass and between the two legume species. Moreover, given known effects of N fertilisation on species diversity effects^37,50^, it was tested whether the N fertilisation treatment interacts with the (pooled) diversity effects, in which case it turned out that interactions of N_Treat with the D_BGL_ term were highly significant, but not with the grass-grass and legume-legume terms, which led to:

3 $${y}_{k}=\sum_{k=1}^{10}\sum_{f=1}^{3}\sum_{i=1}^{4}{\beta }_{ifk}{P}_{i}\times {\mathrm{N}\_\mathrm{Treat}}_{f}\times {\mathrm{FUNC}}_{k}+\sum_{k=1}^{10}\sum_{f=1}^{3}{\delta }_{1fk}{\mathrm{D}}_{\mathrm{BGL}}\times {\mathrm{N}\_\mathrm{Treat}}_{f}\times {\mathrm{FUNC}}_{k}+\sum_{k=1}^{10}{{\delta }_{2k}P}_{\mathrm{Lp}}{P}_{\mathrm{Dg}}\times {\mathrm{FUNC}}_{k}+\sum_{k=1}^{10}{{\delta }_{3k}P}_{\mathrm{Tp}}{P}_{\mathrm{Tr}}\times {\mathrm{FUNC}}_{k}+{\lambda \mathrm{Plot}+\varepsilon }_{k}$$

with Lp: *L. perenne*, Dg: *D. glomerata*, Tp: *T. pratense*, Tr: *T. repens*, and D_BGL_ = *P*_Lp_*P*_Tp_ + *P*_Lp_*P*_Tr_ + *P*_Dg_*P*_Tp_ + *P*_Dg_*P*_Tr_, representing the four pooled pairwise interactions between grass and legume species. All other variables and their related regression coefficients have meanings as explained. The marginal and conditional *R*^2^ (following Nakagawa and Schielzeth^73^) of this regression was 0.876 and 0.881, respectively, which led us to conclude that predictions based on Eq. (3) were highly reliable (see also Supplementary Fig. S6 for observed *versus* predicted values of the ten functions based on Eq. 3, and Supplementary Table S2 for goodness-of-fit measures for selected models).

We choose the four-species equi-proportional mixture as a reference mixture to evaluate beneficial effects on functional performance in mixtures as compared to the average of monocultures (overyielding: OY) using the estimated coefficients of the final model (Eq. 3):

4 $${\mathrm{OY}}_{k} ({\mathrm{\%}})=\frac{{\widehat{y}}_{\mathrm{equi}\_k}- {\widehat{y}}_{\mathrm{avemono}\_k}}{{\widehat{y}}_{\mathrm{avemono}\_k}}\times 100$$

with *ŷ*_equi_*k*_ being the predicted functional performance of function *k* at the four-species equi-proportional mixture and *ŷ*_avemono_*k*_ the predicted performance of the average of monocultures. The 95% confidence interval (CI) to overyielding was calculated by parametric bootstrapping^74^. Because we intended to achieve an approximate multivariate normal distribution of the bootstrap sample, the procedure was performed with the closely related log response ratio (LRR):

5 $${\mathrm{LRR}}_{{\mathrm{equi}}_{k}}=\mathit{ln}\left(\frac{{\widehat{y}}_{\mathrm{equi}\_k}}{{\widehat{y}}_{\mathrm{avemono}\_k}}\right)=ln\left(\frac{{\mathrm{OY}}_{k}}{100}+1\right)$$

with meanings of components as explained, and lastly the LRR was rescaled to OY to give the confidence intervals (see Supplementary Appendix S1 for details to the bootstrap sampling). We note that eqs. (1–5) were also applied to data of each individual year (year 1–3), and we refer to Supplementary Appendix S1 for details of the single years’ analyses.

Finally, the diversity-multifunctionality relationship was evaluated using the mean LRR (MLRR) across all functions. This follows the reasoning that a greater number of functions with higher LRRs indicates enhanced multifunctionality in mixtures as compared to monocultures. To this aim, the LRR as defined in Eq. (5) was generalised to:

6 $${\mathrm{LRR}}_{k}=\mathit{ln}\left(\frac{{\widehat{y}}_{\mathrm{mix}\_k}}{{\widehat{y}}_{\mathrm{ave}\_\mathrm{w}\_\mathrm{mono}\_k}}\right)$$

Here, *ŷ*_mix_*k*_ is the predicted functional performance of function *k* of any mixture and *ŷ*_ave_w_mono_*k*_ the predicted performance of the weighted average of monocultures (all based on Eq. 3), the weights being the species proportions in the mixture. For functions where minimal values were regarded as of positive benefit (SD_yield_, weed biomass, and NO_3_), their LRR was multiplied by − 1. Calculation of LRR_*k*_ was then followed by computation of the MLRR across functions:

7 $${\mathrm{MLRR}}_{\mathrm{D}}=\frac{1}{k}\sum_{k=1}^{10}\mathit{ln}\left(\frac{{\widehat{y}}_{\mathrm{mix}\_k}}{{\widehat{y}}_{\mathrm{ave}\_\mathrm{w}\_\mathrm{mono}\_k}}\right)$$

Note that the MLRR_D_ reflects a *change* in multifunctionality in dependence on the components of the ratio (here: mixtures *versus* averaged monocultures, reflecting a change in species diversity). We prefer such a metric over absolute measures of multifunctionality, as absolute measures are highly context specific and have little value for comparison among systems. To provide a statistical inference to the MLRR, *t* tests against zero were not applicable because the single LRRs must be assumed to be correlated among functions for any given community. Instead, we used generalised least squares regression and implemented the correlation matrix among single LRRs that could be derived from the bootstrap sampling.

Justified by the outcome of Eq. (3), the MLRR_D_ was calculated for a range of overall legume proportions with equal proportions of the two grass and the two legume species to display the effect of species diversity and grass–legume interactions on multifunctionality. The range of legume proportions for which the MLRR_D_ was significantly different from zero was calculated using the Johnson Neyman technique^38^. See Supplementary Appendix S1 for details of the procedure, including the range test.

The MLRR approach is flexible in that it allows the consideration of different comparisons, reflected by the components of the corresponding LRRs. We directly tested the effect of N fertilisation on multifunctionality using comparisons of the four-species reference mixture at N50 with three selected types of communities at N450:

8 $${\mathrm{MLRR}}_{\mathrm{N}}=\frac{1}{k}\sum_{k=1}^{10}\mathit{ln}\left(\frac{{\widehat{y}}_{\mathrm{equi}\_k\_\mathrm{N}50}}{{\widehat{y}}_{\mathrm{community}\_k\_\mathrm{N}450}}\right)$$

where *ŷ*_equi_*k*_N50_ is the predicted functional performance of function *k* at the four-species equi-proportional mixture at N50, and *ŷ*_community_*k*_N450_ is the predicted functional performance of a community at N450 (both based on Eq. 3), namely either (i) the average of the two grass monocultures, (ii) the average of all monocultures, or (iii) the four-species equi-proportional mixture. For functions in which minimal values were targeted, their LRR was multiplied by − 1. See Supplementary Appendix S1 for details of the inference to the MLRR_N_. All analyses were performed using the statistical software R, version 3.6.1^75^ and the package nlme for linear mixed-effects models^76^.

## Supplementary Information

Supplementary Information

## Data Availability

The data generated and analysed during the current study are available in the Dryad Digital Repository at 10.5061/dryad.5dv41ns5d.
