# Supplementary material for: Multispecies for multifunctions: combining four complementary species enhances multifunctionality of sown grassland
Source: Sci Rep. 2021 Feb 15;11:3835. doi: 10.1038/s41598-021-82162-y (PMC7884733; doi:10.1038/s41598-021-82162-y)
Supplement: Supplementary file 1 — Supplementary Information [file 41598_2021_82162_MOESM1_ESM.pdf]

Supplementary Information

Multispecies for multifunctions: combining four complementary species enhances multifunctionality of sown grassland

Matthias Suter, Olivier Huguenin-Elie, Andreas Lüscher

Agroscope, Forage Production and Grassland Systems, Reckenholzstrasse 191, 8046 Zürich, Switzerland

Appendix S1 Supporting text and information on the methods and analyses

Summary of shift of species proportions

The shift of species proportions over the three experimental years is analysed in Nyfeler *et al.*[1]. Here, a summary of the pooled proportions of legume and grass species is given. Overall, legume proportions were significantly affected by N fertilisation, which favoured grasses over legumes.

At N50, observed averaged legume proportions across all four-species mixtures were 42%, 56% and 24% in years 1, 2, and 3, respectively, while averaged grass proportions were 55%, 43% and 73% (the % difference of legume + grass proportions to 100% were unsown species).
At N150, these respective values were 38%, 45%, and 12% for legume species (years 1 to 3, respectively) and 60%, 54%, and 87% for grass species.
At N450, these respective values were 32%, 24%, and 5% for legume species (years 1 to 3, respectively) and 64%, 75%, and 92% for grass species.

Measurement of functions

Symbiotic N_2 fixation

The atom percent excess ^{15}N of the double-labelled ^{15}N-enriched ammonium-nitrate ($^{15}NH_4$ $^{15}NO_3$) was 3.0%, 1.0%, and 0.3% at N50, N150 and N450, respectively. N content was measured i) from bulk samples collected from the permanent sub-plots at each harvest and ii) from each species fraction from sub-plots at the first, third and fifth harvests. Plant samples were dried, ground and milled, and were analysed for their ^{15}N and ^{14}N content by gas isotope ratio mass spectrometry (GIRMS, University of Saskatchewan, Canada) and by thermal conductometry (Dumas and Vario MAX CN, Elementar).

Nitrogen from symbiotic N_2 fixation in the sward comprises legume N directly derived from the atmosphere (N_{dfa}) and grass N derived from apparent transfer (N_{trans}) of N_{dfa}. Thus:
$N_{sym} = N_{dfa} + N_{trans}$

Nitrogen derived from the atmosphere (N_{dfa}) in the legume species was calculated following McAuliffe *et al.*[2]. Values of excess ^{15}N relative to atmospheric N_2 were compared to those of a non-fixing reference plant:

$$N_{dfa}\ (\%) = \left(1 - \frac{^{15}\text{N atom\% excess in legume}}{^{15}\text{N atom\% excess in reference}}\right) \times 100 \qquad \text{(S1)}$$

with the legume being either *T. pratense* or *T. repens,* and *L. perenne* grown in the same plot acting as the reference plant.

Apparent N transfer from legume to grass species was calculated following Vallis *et al.*[3]:

$$N_{trans}\,(\%) = \left(1 - \frac{^{15}\text{N atom\% excess in grassmix}}{^{15}\text{N atom\% excess in grassmono}}\right) \times 100 \qquad \text{(S2)}$$

where 'grassmix' are the grass species grown in mixtures, and 'grassmono' are the grass species grown in monocultures adjacent to the mixture plot and at the same N level. The amount of N (kg N ha^{-1} yr^{-1}) of N_{dfa} and N_{trans} was calculated using the N content in the species and their respective dry mass. The two fractions N_{dfa} and N_{trans} were summed to give N_{sym}. See Nyfeler *et al.*[4] for full details of calculations and a critical appraisal of the methods.

NO_3 in soil solution

Sampling plots for NO_3 in soil solution were monocultures, the four-species equi-proportional mixture, and the dominant mixtures (Table S1, 54 plots in total). In each sampling plot, a porous cup tension lysimeter was used to collect samples for nitrate-N (NO_3-N) analysis. These lysimeters were composed of two porous ceramic suction cups installed at depth of 60 cm and a horizontal distance of 1.4 m. Suction cups were connected to an air-tight polymer tube and the two tubes per plot were connected to each other to obtain bulk soil solution samples. In two-week intervals, a suction of 80 kPa was applied one day prior to sampling; thus, samples represented a 24-hour composite. Samples were deep-frozen within less than two hours after their collection. Concentration of NO_3-N was determined from defrosted samples by spectrophotometry using a Segmented Flow Analyser (Skalar SANplus, Skalar Analytical, Breda). Sampling took place from October 2004 to April 2006. Because sampled soil solutions dropped close to zero during the dry summer months, we defined two periods from 19.10.2004 - 13.04.2005 and from 17.10.2005 - 07.04.2006, over which measured values were averaged for statistical analyses. These two periods are referred to as year 2 and 3.

Organic matter digestibility

Yield samples (0.5 g) from sub-plots of each of the second and fourth harvest were incubated in strained rumen liquor for 48 hours at 40 °C, followed by further digestion in acidified pepsin solution for another 48 hours at 40 °C. The indigestible residue, from which the inorganic fraction was first subtracted, was weighted and scaled to dry matter. The organic matter of the sample (received by dry matter combustion at 600 °C to constant weight) minus the

indigestible residue resulted in OM digestibility. For each analytical batch, the activity of the microbial inoculum was normalized by an identical treatment of 20 to 30 samples of reference material with known (*in vivo*) digestibility. Values of the second and the fourth harvest were averaged to determine OM digestibility for a certain year.

Metabolisable energy

Metabolisable energy content (ME) is defined following a reference manual of Agroscope[5] in a two-stage process. First, digestible crude protein (DCP) is determined using an empirical scaling equation based on a large number of samples with:

$$DCP = CP \times (0.33 + 0.0033 \times CP_{OS} - 0.0000061 \times CP_{OS}^2) \qquad (S3)$$

where CP denotes crude protein content and CP_{OS} denotes crude protein content per organic matter (OM) (i.e. CP_{OS} = g CP/kg OM). CP and OM are determined as described in the main text.

ME is then computed as:

$$ME = 0.0142 \times OMD + 0.0059 \times DCP, \quad \text{if } OMD/DCP < 7$$
$$ME = 0.0151 \times OMD, \quad \text{if } OMD/DCP \geq 7 \qquad (S4)$$

Data analyses

The univariate diversity interaction model

Kirwan *et al.*[6] introduced the univariate diversity interaction model using multiple linear regression. The approach allows modelling of the response variable as a function of species proportions, the latter summing to unity in each stand (see also Cornell[7] for extensive information). The community-level response is modelled as a linear combination of (1) identity effects of species as given by their monoculture performance, (2) species net interactions, termed diversity effects (*D*: being positive, negative or neutral), which are defined as the difference between the actual mixture performance and that expected from the relative contribution of the constituent monocultures, and (3) any further variables, such as overall sowing density or N fertilisation. Given this framework, a basic univariate diversity interaction model to one function of our data is:

$$y = \alpha\text{DENS} + \sum_{i=1}^{4} \beta_i P_i + \gamma\text{N\_Treat} + \sum_{\substack{i,j=1 \\ i<j}}^{4} \delta_{ij} P_i P_j + \varepsilon$$

(S5)

The α coefficient denotes the effect of changing the overall sowing density on the response variable y, e.g. yield. With P_i denoting the species' proportions in a stand, coefficients β_1 to β_4 estimate the effects of species' proportional contributions on y and, if $P = 1$, β coefficients estimate the response y of species' monocultures. The γ coefficient estimates the effect of N fertilisation, with N_Treat being a factor with three levels: N50, N150, and N450. Coefficients δ_1 to δ_6 estimate the six possible pairwise interactions among the four species to model diversity effects. The residual term ε is assumed to be normally distributed with constant variance σ^2. Interactions between e.g. N_Treat and P_i can be added to eqn. S1.

Bootstrap sampling

Parametric bootstrapping was based on the estimated coefficients of the final model (eqn. 3 main text) and related residual variances of functions with corresponding co-variance matrix Σ. Because residuals were correlated, we randomly sampled from a multivariate normal distribution with a zero-vector of means and given the residual variances and Σ, as estimated by eqn. (3). This allowed for calculation of new data (fitted values from model + randomly sampled 'residuals') to which eqn. (3) was fitted, followed by calculation of the log response ratio (LRR) and overyielding as defined in the main text. The procedure was repeated 1000 times. Functions that were natural log transformed in eqn. (3), namely stability, weed biomass and NO_3 in soil solution, were back-transformed to linear scale before calculating the LRR and overyielding.

Analyses of single year's data

Analyses of the data of each experimental year followed the same principles as the data averaged across years (eqs. 1-5 main text). The following details need to be mentioned:

Regarding the variable P_i, which denotes species proportions in a stand (eqs. 1-3), the sowing proportions were used as a predictor for analyses in year 1, whereas observed species proportions in annual biomass yield of the preceding year were used in years 2 and 3. Using the previous year's species proportions as predictors was done to overcome effects of year-to-year changes in community composition.

In all analyses of single years, the functions weed biomass and NO_3 were first natural log transformed and then scaled to range between 0 and 100% to achieve a multivariate normal residual distribution.

Regarding sowing density, increased density only had an effect in year 1 and only on weed biomass. Consequently, the variable sowing density was omitted from the models for years 2 and 3. At year 1, increased sowing density resulted in 24% less weed biomass in the four-species equi-proportional reference mixture at all N fertilisation treatments (t_{361} = 2.45, P = 0.015). Notably, the weed reduction occurred on low absolute levels (Fig. S2) and resulted in 0.19, 0.20, and 0.20 t weed biomass ha^{-1} yr^{-1} in the reference mixture at N50, N150, and N450, respectively, at high sowing density. Regarding all other functions, the effect of sowing density was not significant ($t < 1.65$, $P > 0.1$).

To increase parsimony in the regression equation, pooling of pairwise species interactions and hierarchical testing of nested models was performed as described in the main text for the analysis across years. It was revealed that a model as specified by eqn. (3) (including sowing density in year 1) was the most appropriate model to analyse the data of each experimental year. This outcome also allowed the direct comparison of parameter estimates and predictions among single years and across years. The marginal R^2 (following Nakagawa and Schielzeth[8]) of the final regressions (eqn. 3) ranged between 0.896 (year 1) and 0.876 (year 2), while the conditional R^2 ranged between 0.900 (year 1) and 0.878 (year 2), indicating high reliability of model predictions also for individual years.

Test of multifunctionality ($MLLR_D$) over a range of legume proportions

We wished to calculate the inference to the $MLRR_D$ across functions for a range of legume proportions (see Fig. 3). While the $MLRR_D$ over the range of legume proportions could be derived from predictions based on eqn. (3), bootstrap sampling at incremental steps (to calculate the correlations among individual functions' LRR, needed for the inference to the $MLRR_D$) was not feasible. Instead, we performed a bootstrap sampling (as described) at overall legume proportions of 0, 0.05, 0.15, 0.25, 0.37, 0.50, 0.63, 0.75, 0.85, 0.95, and 1 in mixtures, with equal proportions of the two grass and the two legume species. The denominator of individual functions' LRR was the predicted functional response at the weighted average of monocultures, with the weights being the species' proportions in the mixtures. These bootstrap samples provided a multivariate normal distribution of individual functions' LRR, which allowed calculation of correlations among them. The correlation matrix among functions' LRR

at intermediate legume proportions was then computed as the weighted average between each of two 'neighbouring' correlation matrices (e.g., between 0.05 and 0.15 legume proportion, 0.15 and 0.25 legume proportion). It turned out that the mean absolute difference between correlations of each of two 'neighbouring' matrices was only 0.044, 0.040, and 0.044 at N50, N150, and N450, respectively, which strongly justified this approach. The correlations among individual functions' LRR were then implemented as a fixed model matrix defining the residual correlation in the generalised least square regressions to test the $MLRR_D$ against zero (using the gls function from the nlme package of the R software), which provided also the variance of the estimated difference. Finally, variance and difference were needed to calculate the range of legume proportions, for which the $MLRR_D$ was significantly different from zero (following Johnson and Neyman[9]; Potthoff[10]: point-wise test).

Effect of log transformation of functions on multifunctionality

We further evaluated the effect of the natural log transformation of the functions stability, weed biomass, and NO_3 in soil solution on the outcome of the $MLLR_D$. Log transformation of data in regression analyses can introduce bias in that the geometric mean is predicted on the back-transformed linear scale, the geometric mean being always equal or smaller than the linear mean of untransformed data. Because the $MLLR_D$ as defined in eqn. (7), main text, is a mean of several log response ratios, the effect of log transformation of single functions on the $MLLR_D$ is not directly obvious. To assess this aspect, we computed the functional responses and the $MLLR_D$ as described in the main text but left the functions stability, weed biomass, and NO_3 untransformed. We calculated the $MLLR_D$ for overall legume proportions of 0, 0.25, 0.5, 0.75 and 1 in mixtures, at the three N fertilisation treatments. It turned out that the regression with untransformed data resulted in (unrealistic) negative mixture predictions for weed biomass and NO_3 in one fifth of cases, which made a comparison of the $MLRR_D$ impossible in these situations. Where a comparison was possible, the $MLRR_D$ based on the regression with untransformed data was mostly larger than or equal to the $MLRR_D$ calculated with the three functions log transformed. We thus concluded that our statements regarding the diversity-multifunctionality relationship and the resource use-multifunctionality relationship were generally conservative.

Test of the effect of N fertilisation on multifunctionality ($MLLR_N$)

The effect of N fertilisation on multifunctionality was tested by calculating the individual functions' LRR as defined by eqn. (8) followed by performing a bootstrap sampling taking

1000 samples (as described above). This allowed the calculation of the correlation matrix among single LRRs, which was implemented in a generalised least square regression to test the $MLRR_N$ against zero. We performed three distinct comparisons related to N fertilisation: four-species equi-proportional mixture at N50 against i) the average of the two grass monocultures at N450, ii) the average of all monocultures at N450, iii) the four-species equi-proportional mixture at N450; therefore, three bootstrap runs were performed, one for each case.

References

1. Nyfeler, D. *et al.* Strong mixture effects among four species in fertilized agricultural grassland led to persistent and consistent transgressive overyielding. *J. Appl. Ecol.* **46,** 683–691 (2009).
2. McAuliffe, C., Chamblee, D. S., Uribe-Arango, H. & Woodhouse, W. W. Influence of inorganic nitrogen on nitrogen fixation by legumes as revealed by ^{15}N. *Agron. J.* **50**, 334–337 (1958).
3. Vallis, I., Henzell, E. F. & Evans, T. R. Uptake of soil-nitrogen by legumes in mixed swards. *Aust. J. Agric. Res.* **28**, 413–425 (1977).
4. Nyfeler, D., Huguenin-Elie, O., Suter, M., Frossard, E. & Lüscher, A. Grass-legume mixtures can yield more nitrogen than legume pure stands due to mutual stimulation of nitrogen uptake from symbiotic and non-symbiotic sources. *Agr. Ecosyst. Environ.* **140**, 155–163 (2011).
5. Agroscope. *Fütterungsempfehlungen für Wiederkäuer* (Grünes Buch) (Agroscope, Posieux, Schweiz, 2013).
6. Kirwan, L. *et al.* Diversity-interaction modeling: estimating contributions of species identities and interactions to ecosystem function. *Ecology* **90**, 2032–2038 (2009).
7. Cornell, J. A. *Experiments with mixtures* (Wiley, New York, 2002).
8. Nakagawa, S. & Schielzeth, H. A general and simple method for obtaining R^2 from generalized linear mixed-effects models. *Methods Ecol. Evol.* **4** 133–142 (2013).
9. Johnson, P. O. & Neyman, J. Tests of certain linear hypotheses and their application to some educational problems. *Stat. Res. Memoirs* **1**, 57–93 (1936).
10. Potthoff, R. F. (2006) Johnson-Neyman technique in *Encyclopedia of Statistical Sciences*, vol 6 (ed Kotz, S.) 3745–3749 (Wiley, 2006).

Appendix S2 Supplementary Tables and Figures

Table S1. Species proportions in monocultures and mixtures sown following a simplex design. All communities were sown at two overall densities and subjected to three levels of N fertiliser application (50, 150, 450 kg N ha^{-1} yr^{-1}), except the binary and co-dominant mixtures, which were established only at 150 kg N ha^{-1} yr^{-1}.

Stand	*L. perenne*	*D. glomerata*	*T. pratense*	*T. repens*
Monocultures	1	0	0	0
	0	1	0	0
	0	0	1	0
	0	0	0	1
Equi-proportional mixture	0.25	0.25	0.25	0.25
Dominant mixtures	0.70	0.10	0.10	0.10
	0.10	0.70	0.10	0.10
	0.10	0.10	0.70	0.10
	0.10	0.10	0.10	0.70
Binary mixtures	0.50	0.50	0	0
	0.50	0	0.50	0
	0.50	0	0	0.50
	0	0.50	0.50	0
	0	0.50	0	0.50
	0	0	0.50	0.50
Co-dominant mixtures	0.40	0.40	0.10	0.10
	0.40	0.10	0.40	0.10
	0.40	0.10	0.10	0.40
	0.10	0.40	0.40	0.10
	0.10	0.40	0.10	0.40
	0.10	0.10	0.40	0.40

Table S2. Goodness-of-fit measures for selected multivariate mixed-effects models regressing the responses of ten function on species proportions and three N treatments. See materials and methods, main text, for definition and interpretation of model terms. Additional models to those described in the main text are listed.

ID	Model (only fixed effects listed)	R^2_m‡	R^2_c‡	LogLik§	AIC§	Remarks
A	$\sum_{k=1}^{10} \mathrm{FUNC}_k$	0.519	0.533	808.1	-1484.2	Baseline model; R^2 captures differences among functions across species proportions and N levels
B	$\sum_{k=1}^{10}\sum_{i=1}^{4} P_i \times \mathrm{FUNC}_k$	0.650	0.655	972.5	-1753.0	Species' identity effects for the ten functions, across N levels
C	$\sum_{k=1}^{10}\sum_{f=1}^{3}\sum_{i=1}^{4} P_i \times \mathrm{N\_Treat}_f \times \mathrm{FUNC}_k$	0.788	0.793	1235.2	-2218.5	Species' identity effects for the ten functions at the three N levels
D	$\sum_{k=1}^{10}\sum_{f=1}^{3}\sum_{i=1}^{4} P_i \times \mathrm{N\_Treat}_f \times \mathrm{FUNC}_k + \sum_{k=1}^{10}\sum_{\substack{i,j=1\\i<j}}^{4} P_iP_j \times \mathrm{FUNC}_k$	0.867	0.872	1345.8	-2225.5	Eqn. 2, main text, yet without effect of sowing density (which was not significant); three P_iP_j terms for NO_3 in soil solution omitted due to singularity in the design matrix
E	$\sum_{k=1}^{10}\sum_{f=1}^{3}\sum_{i=1}^{4} P_i \times \mathrm{N\_Treat}_f \times \mathrm{FUNC}_k + \sum_{k=1}^{10} \mathrm{D_{BGL}} \times \mathrm{FUNC}_k + \sum_{k=1}^{10} P_{\mathrm{Lp}}P_{\mathrm{Dg}} \times \mathrm{FUNC}_k + \sum_{k=1}^{10} P_{\mathrm{Tp}}P_{\mathrm{Tr}} \times \mathrm{FUNC}_k$	0.865	0.870	1325.9	-2243.7	Pooling of four individual P_iP_j terms to D_{BGL}, the latter modelling pooled interactions between grass and legume proportions¶; $P_{Lp}P_{Dg}$ and $P_{Tp}P_{Tr}$ for NO_3 omitted due to singularity in the design matrix
F	$\sum_{k=1}^{10}\sum_{f=1}^{3}\sum_{i=1}^{4} P_i \times \mathrm{N\_Treat}_f \times \mathrm{FUNC}_k + \sum_{k=1}^{10} P_{\mathrm{Lp}}P_{\mathrm{Dg}} \times \mathrm{FUNC}_k + \sum_{k=1}^{10} P_{\mathrm{Tp}}P_{\mathrm{Tr}} \times \mathrm{FUNC}_k$	0.806	0.810	1265.1	-2142.2	Intermediate model to evaluate D_{BGL}×FUNC (against E), and D_{BGL}×N_Treat×FUNC (against J); $P_{Lp}P_{Dg}$ and $P_{Tp}P_{Tr}$ for NO_3 omitted due to consistency
G	$\sum_{k=1}^{10}\sum_{f=1}^{3}\sum_{i=1}^{4} P_i \times \mathrm{N\_Treat}_f \times \mathrm{FUNC}_k + \sum_{k=1}^{10} \mathrm{D_{BGL}} \times \mathrm{FUNC}_k$	0.854	0.860	1293.2	-2214.5	Intermediate model to evaluate $P_{Lp}P_{Dg}$×FUNC and $P_{Tp}P_{Tr}$×FUNC (against E)
H	$\sum_{k=1}^{10}\sum_{f=1}^{3}\sum_{i=1}^{4} P_i \times \mathrm{N\_Treat}_f \times \mathrm{FUNC}_k + \sum_{k=1}^{10}\sum_{f=1}^{3} \mathrm{D_{BGL}} \times \mathrm{N\_Treat}_f \times \mathrm{FUNC}_k$	0.869	0.874	1367.8	-2323.6	Intermediate model to evaluate the interaction of D_{BGL}×FUNC with N_Treat (against G)
J	$\sum_{k=1}^{10}\sum_{f=1}^{3}\sum_{i=1}^{4} P_i \times \mathrm{N\_Treat}_f \times \mathrm{FUNC}_k + \sum_{k=1}^{10}\sum_{f=1}^{3} \mathrm{D_{BGL}} \times \mathrm{N\_Treat}_f \times \mathrm{FUNC}_k + \sum_{k=1}^{10} P_{\mathrm{Lp}}P_{\mathrm{Dg}} \times \mathrm{FUNC}_k + \sum_{k=1}^{10} P_{\mathrm{Tp}}P_{\mathrm{Tr}} \times \mathrm{FUNC}_k$	0.876	0.881	1392.0	**-2335.9**	**Eqn. 3, main text. Final model**. $P_{Lp}P_{Dg}$ and $P_{Tp}P_{Tr}$ for NO_3 omitted due to singularity in the design matrix. Likelihood ratio test for reasonable comparisons (against H, F, E, C) highly significant (**$\chi^2 > 48.2$, $P \leq 0.0001$**)

‡ R^2_m and R^2_c: marginal and conditional R^2, respectively, following Nakagawa and Schielzeth (2013). Corresponding model used restricted maximum likelihood. R^2_m: variance explained by fixed effects; R^2_c: variance explained by fixed and random effects.

§ LogLik: log likelihood; AIC: Akaike Information Criterion. Corresponding model used maximum likelihood.

¶ $D_{BGL} = P_{Lp}P_{Tp} + P_{Lp}P_{Tr} + P_{Dg}P_{Tp} + P_{Dg}P_{Tr}$

Nakagawa, S. & Schielzeth, H. A general and simple method for obtaining R^2 from generalized linear mixed-effects models. *Methods in Ecology and Evolution* **4,** 133–142 (2013).

Table S3. Predicted performance of seven functions in monocultures of four forage species and the four-species equi-proportional mixture at three N fertilisation treatments in **year 1** (N50: 50 kg N ha^{-1} yr^{-1}, N150: 150 kg N ha^{-1} yr^{-1}, N450: 450 kg N ha^{-1} yr^{-1}). Values are for average sowing density and are in % of the maximal performance per function (max) at a single year over the three-year experiment and N fertilisation treatments.

Function	Maximal performance per function (max)	N treatment	Monocultures[‡] Performance (% of max)					Equi-proportional mixture Performance (% of max)	Diversity effects[§] (% of max)		
			Lp	Dg	Tp	Tr	Average		D_{BGL} effect	Lp·Dg effect	Tp·Tr effect
Yield	19.96	N50	36.9	35.1	71.8	61.2	51.2	79.9	24.7***	2.6*	1.3^{ns}
	(t ha^{-1} yr^{-1})	N150	40.9	41.7	78.2	54.2	53.7	81.7	24.1***	2.6*	1.3^{ns}
		N450	54.0	55.5	74.5	64.0	62.0	88.5	22.7***	2.6*	1.3^{ns}
		s.e.[¶]	4.23	4.23	4.23	4.23	2.38	2.49	3.79	1.25	1.25
Seasonal	3.24	N50	60.5	30.9	58.0	43.7	48.3	71.8	17.3**	2.7[†]	3.6*
SD_{Yield}	(t ha^{-1} yr^{-1})	N150	50.8	25.5	54.2	34.8	41.3	68.3	20.7***	2.7[†]	3.6*
		N450	41.3	20.5	55.6	45.7	40.8	71.2	24.2***	2.7[†]	3.6*
		s.e.	4.83	4.83	4.83	4.83	2.72	2.84	4.33	1.42	1.42
Seasonal stability	18.27	N50	21.7	39.1	41.6	48.1	37.6	37.3	4.5^{ns}	-2.5*	-2.4*
		N150	32.0	59.9	49.2	53.1	48.5	39.0	-4.6*	-2.5*	-2.4*
		N450	45.7	84.9	46.3	50.3	56.8	40.0	-12.0**	-2.5*	-2.4*
		s.e.	3.60	3.60	3.60	3.60	2.06	2.12	3.23	1.06	1.06
Weed biomass	-[#]	N50	54.3	77.4	63.4	87.1	70.6	62.9	-5.6^{ns}	-0.4^{ns}	-1.7^{ns}
		N150	47.9	74.8	71.6	81.3	68.9	63.7	-3.1^{ns}	-0.4^{ns}	-1.7^{ns}
		N450	52.7	78.0	65.5	80.8	69.2	63.8	-3.3^{ns}	-0.4^{ns}	-1.7^{ns}
		s.e.	3.86	3.86	3.86	3.86	2.17	2.27	3.45	1.14	1.14
N_{sym}	374.4	N50	0.9	4.7	81.2	75.5	40.6	60.8	18.7*	2.2^{ns}	-1.2^{ns}
	(kg ha^{-1} yr^{-1})	N150	<0.1	5.4	91.5	59.7	39.0	57.6	17.6***	2.2^{ns}	-1.2^{ns}
		N450	<0.1	2.1	67.2	53.2	30.5	41.1	9.6^{ns}	2.2^{ns}	-1.2^{ns}
		s.e.	6.55	6.55	6.55	6.55	3.68	3.85	5.87	1.93	1.93

N efficiency	10.54	N50	24.2	30.7	84.6	87.2	56.7	82.3	25.3***	0.9^{ns}	-0.6^{ns}
		N150	9.5	12.5	34.1	25.9	20.5	28.3	7.6***	0.9^{ns}	-0.6^{ns}
		N450	5.2	6.8	11.2	10.7	8.5	10.7	2.0^{ns}	0.9^{ns}	-0.6^{ns}
		s.e.	3.15	3.15	3.15	3.15	1.77	1.85	2.83	0.9	0.9
Crude protein content	322.1 (g kg^{-1} DM)	N50	34.0	43.4	60.0	72.3	52.4	53.3	3.2^{ns}	-0.1^{ns}	-2.3***
		N150	38.9	46.1	67.4	70.9	55.8	53.2	-0.3^{ns}	-0.1^{ns}	-2.3***
		N450	47.3	57.7	68.3	75.7	62.2	55.2	-4.7†	-0.1^{ns}	-2.3***
		s.e.	2.13	2.13	2.13	2.13	1.20	1.25	1.91	0.63	0.63

*** $P \leq 0.001$, ** $P \leq 0.01$, * $P \leq 0.05$, † $P \leq 0.1$, ns: not significant

‡ Lp: *L. perenne*, Dg: *D. glomerata*, Tp: *T. pratense*, Tr: *T. repens*

§ The diversity effect is calculated as the difference between the performance of the equi-proportional four-species mixture and the average of monocultures and is split into the effects of mixing grass and legume species (D_{BGL} effect), mixing the two grasses Lp and Dg (Lp·Dg effect), and mixing the two legumes Tp and Tr (Tp·Tr effect).

¶ Standard errors (s.e.) were calculated from the weighted average of variances at each N fertilisation level.

# Weed biomass was natural log transformed and subsequently scaled to range between 0 and 100%; thus, scaling with the maximal functional performance is not reasonably possible. See Fig. S2 for back-transformed values on the linear scale.

Table S4. Predicted performance of ten functions in monocultures of four forage species and the four-species equi-proportional mixture at three N fertilisation treatments in **year 2** (N50: 50 kg N ha^{-1} yr^{-1}, N150: 150 kg N ha^{-1} yr^{-1}, N450: 450 kg N ha^{-1} yr^{-1}). Values are in % of the maximal performance per function (max) at a single year over the three-year experiment and N fertilisation treatments.

Function	Maximal performance per function (max)	N treatment	Monocultures[‡]					Equi-proportional mixture			
			Performance (% of max)					Performance	Diversity effects[§] (% of max)		
			Lp	Dg	Tp	Tr	Average	(% of max)	D_{BGL} effect	Lp·Dg effect	Tp·Tr effect
Yield	19.96	N50	22.8	30.9	40.3	48.7	35.7	78.2	41.2***	0.9^{ns}	0.3^{ns}
	(t ha^{-1} yr^{-1})	N150	39.0	52.7	52.8	45.3	47.5	83.9	35.2***	0.9^{ns}	0.3^{ns}
		N450	70.4	77.5	57.7	48.0	63.4	87.6	22.9***	0.9^{ns}	0.3^{ns}
		s.e.[¶]	3.60	3.93	4.44	4.87	2.24	2.77	3.50	1.32	2.05
Seasonal	3.24	N50	23.2	16.0	35.1	32.8	26.8	48.3	20.8***	0.8^{ns}	-0.1^{ns}
SD_{Yield}	(t ha^{-1} yr^{-1})	N150	29.6	22.3	41.4	33.7	31.8	55.1	22.7***	0.8^{ns}	-0.1^{ns}
		N450	62.5	28.7	42.3	30.2	40.9	51.8	10.2*	0.8^{ns}	-0.1^{ns}
		s.e.	4.06	4.51	4.80	5.25	2.48	3.03	3.83	1.48	2.25
Seasonal	18.27	N50	32.0	67.5	38.8	50.5	47.2	58.9	13.4***	-1.9†	0.1^{ns}
Stability		N150	47.3	73.5	42.7	46.4	52.5	52.6	1.8^{ns}	-1.9†	0.1^{ns}
		N450	39.5	91.6	45.7	54.4	57.8	58.5	2.5^{ns}	-1.9†	0.1^{ns}
		s.e.	3.27	3.70	3.56	3.91	1.92	2.26	2.91	1.12	1.65
Weed biomass	-[#]	N50	42.4	30.4	95.9	79.8	62.1	46.5	-11.3*	-1.1^{ns}	-3.3^{ns}
		N150	46.6	47.2	97.7	82.0	68.4	42.0	-22.0***	-1.1^{ns}	-3.3^{ns}
		N450	47.3	73.9	92.6	71.9	71.4	57.6	-9.4†	-1.1^{ns}	-3.3^{ns}
		s.e.	5.05	5.70	5.49	6.00	2.96	3.50	4.49	1.76	2.57
N_{sym}	374.4	N50	8.9	2.8	34.7	73.6	30.0	93.1	65.2***	-0.3^{ns}	-1.8^{ns}
	(kg ha^{-1} yr^{-1})	N150[‡‡]	0	0	53.9	59.7	28.4	79.3	53.0***	-0.3^{ns}	-1.8^{ns}
		N450	1.0	<0.1	17.5	30.2	12.1	36.2	26.2**	-0.3^{ns}	-1.8^{ns}
		s.e.	10.23	10.71	9.59	10.55	4.62	5.88	7.33	2.63	4.30

N efficiency	10.54	N50	22.2	24.1	50.1	88.6	46.3	101.8	56.4***	-0.3^{ns}	-0.5^{ns}
		N150	11.7	13.3	24.3	28.8	19.5	33.7	15.1***	-0.3^{ns}	-0.5^{ns}
		N450	8.4	9.7	7.9	11.0	9.2	13.3	4.9^{ns}	-0.3^{ns}	-0.5^{ns}
		s.e.	3.67	4.01	4.48	5.03	2.30	2.74	3.51	1.18	1.88
NO_3	-$^{\#}$	N50	20.3	23.9	59.6	84.6	47.1	42.7	0.4^{ns}	4.1^{ns}	-8.9^{ns}
soil solution		N150	32.5	30.7	74.3	80.7	54.6	32.8	-17.0†	4.1^{ns}	-8.9^{ns}
		N450	57.0	48.7	84.5	97.7	72.0	65.9	-1.3^{ns}	4.1^{ns}	-8.9^{ns}
		s.e.	10.09	10.75	11.19	11.79	5.80	7.83	11.01	4.50	8.30
Crude protein	322.1	N50	42.8	38.7	62.3	92.0	58.9	71.7	11.1***	-0.4^{ns}	2.1^{ns}
content	(g kg^{-1} DM)	N150	45.4	40.0	71.3	95.7	63.1	66.2	1.5^{ns}	-0.4^{ns}	2.1^{ns}
		N450	60.7	56.2	72.6	99.4	72.2	70.7	-3.1^{ns}	-0.4^{ns}	2.1^{ns}
		s.e.	2.76	3.07	3.21	3.56	1.67	2.03	2.58	0.95	1.49
OM digestibility	671.0	N50	95.9	90.9	86.0	95.6	92.1	89.7	-2.1^{ns}	-0.1^{ns}	-0.1^{ns}
	(g kg^{-1} DM)	N150	93.8	92.7	88.2	93.5	92.1	91.5	-0.3^{ns}	-0.1^{ns}	-0.1^{ns}
		N450	96.9	92.2	86.6	96.4	93.0	92.6	-0.1^{ns}	-0.1^{ns}	-0.1^{ns}
		s.e.	1.41	1.61	1.57	1.78	0.85	0.96	1.26	0.43	0.64
Metabolisable	10.77	N50	92.0	85.8	85.1	96.9	89.9	90.3	-1.2^{ns}	-0.1^{ns}	1.7^{ns}
energy	(MJ kg^{-1} DM)	N150	89.5	87.4	88.0	95.5	90.1	91.3	-0.3^{ns}	-0.1^{ns}	1.7^{ns}
		N450	95.3	89.5	87.1	98.1	92.5	92.8	-1.3^{ns}	-0.1^{ns}	1.7^{ns}
		s.e.	1.33	1.52	1.50	1.69	0.81	0.93	1.21	0.43	0.63

*** $P \leq 0.001$, ** $P \leq 0.01$, * $P \leq 0.05$, † $P \leq 0.1$, ns: not significant

‡ Lp: *L. perenne*, Dg: *D. glomerata*, Tp: *T. pratense*, Tr: *T. repens*

§ The diversity effect is calculated as the difference between the performance of the equi-proportional four-species mixture and the average of monocultures and is split into the effects of mixing grass and legume species (D_{BGL} effect), mixing the two grasses Lp and Dg (Lp·Dg effect), and mixing the two legumes Tp and Tr (Tp·Tr effect).

¶ Standard errors (s.e.) were calculated from the weighted average of variances at each N fertilisation level.

$^{\#}$ Weed biomass and NO_3 in soil solution were natural log transformed and subsequently scaled to range between 0 and 100%; thus, scaling with the maximal functional performance is not reasonably possible. See Fig. S3 for back-transformed values on the linear scale.

‡‡Parameters for monocultures of Lp and Dg at N150 were held constant at zero to avoid the estimation of negative N_{sym} values.

Table S5. Predicted performance of ten functions in monocultures of four forage species and the four-species equi-proportional mixture at three N fertilisation treatments in **year 3** (N50: 50 kg N ha^{-1} yr^{-1}, N150: 150 kg N ha^{-1} yr^{-1}, N450: 450 kg N ha^{-1} yr^{-1}). Values are in % of the maximal performance per function (max) at a single year over the three-year experiment and N fertilisation treatments.

Function	Maximal performance per function (max)	N treatment	Monocultures[‡] Performance (% of max)					Equi-proportional mixture Performance	Diversity effects[§] (% of max)		
			Lp	Dg	Tp	Tr	Average	(% of max)	D_{BGL} effect	Lp·Dg effect	Tp·Tr effect
Yield	19.96	N50	27.4	31.7	37.5	38.6	33.8	63.8	27.2***	2.9**	-0.1^{ns}
	(t ha^{-1} yr^{-1})	N150	41.0	46.7	46.6	39.2	43.4	69.0	22.8***	2.9**	-0.1^{ns}
		N450	65.2	78.0	48.2	41.1	58.1	69.7	8.8[†]	2.9**	-0.1^{ns}
		s.e.[¶]	4.40	4.32	4.52	4.73	2.39	2.64	3.72	1.03	1.46
Seasonal	3.24	N50	29.8	18.3	33.0	32.4	28.4	60.4	30.0***	1.7^{ns}	0.4^{ns}
SD_{Yield}	(t ha^{-1} yr^{-1})	N150	35.2	21.8	35.3	31.9	31.1	61.6	28.5***	1.7^{ns}	0.4^{ns}
		N450	61.4	70.1	44.1	30.7	51.6	87.9	34.3***	1.7^{ns}	0.4^{ns}
		s.e.	5.12	5.01	5.27	5.45	2.76	3.06	4.30	1.25	1.72
Seasonal	18.27	N50	31.2	56.8	39.1	40.9	42.0	36.8	-6.0*	1.1^{ns}	-0.3^{ns}
Stability		N150	41.4	61.0	44.3	41.5	47.0	38.2	-9.7***	1.1^{ns}	-0.3^{ns}
		N450	35.1	38.0	36.9	44.9	38.7	27.3	-12.2***	1.1^{ns}	-0.3^{ns}
		s.e.	3.04	2.99	3.10	3.21	1.63	1.80	2.53	0.78	1.03
Weed biomass	-[#]	N50	70.5	44.8	94.9	54.8	66.2	53.4	-11.4^{ns}	-1.8^{ns}	0.3^{ns}
		N150	66.2	50.8	89.9	55.2	65.5	34.0	-29.9***	-1.8^{ns}	0.3^{ns}
		N450	43.0	31.5	88.8	47.7	52.8	65.0	13.7^{ns}	-1.8^{ns}	0.3^{ns}
		s.e.	12.3	12.1	12.6	13.1	6.62	7.37	10.3	3.15	4.21
N_{sym}	374.4	N50	3.6	<0.1	22.0	33.1	14.5	32.4	17.8***	-[‡‡]	-
	(kg ha^{-1} yr^{-1})	N150	1.3	<0.1	25.9	27.7	12.8	28.5	15.8***	-	-
		N450	2.2	<0.1	19.1	18.8	9.9	22.0	12.1*	-	-
		s.e.	4.93	4.95	4.98	5.09	2.60	2.66	3.91	-	-

N efficiency	10.54	N50	22.9	22.9	43.6	57.3	36.7	57.2	19.1***	0.3^{ns}	1.1^{ns}
		N150	12.4	12.7	19.0	18.3	15.6	22.6	5.6**	0.3^{ns}	1.1^{ns}
		N450	9.0	10.5	7.2	8.0	8.7	11.0	0.9^{ns}	0.3^{ns}	1.1^{ns}
		s.e.	3.12	3.09	3.19	3.32	1.68	1.84	2.60	0.74	1.03
NO_3	-[#]	N50	19.0	21.3	61.1	75.5	44.2	29.8	-14.5*	-[‡‡]	-
soil solution		N150	34.1	11.4	56.0	84.4	46.5	32.9	-13.6*	-	-
		N450	77.9	85.7	87.4	98.3	87.3	93.6	6.3^{ns}	-	-
		s.e.	8.39	8.41	8.43	8.50	4.39	4.74	6.83	-	-
Crude protein	322.1	N50	42.1	37.5	58.6	75.7	53.5	45.5	-7.8**	-0.7^{ns}	0.6^{ns}
content	(g kg^{-1} DM)	N150	44.1	42.6	64.0	76.2	56.7	50.5	-6.0***	-0.7^{ns}	0.6^{ns}
		N450	66.4	60.6	68.9	87.9	70.9	68.3	-2.5^{ns}	-0.7^{ns}	0.6^{ns}
		s.e.	2.69	2.63	2.76	2.86	1.45	1.60	2.24	0.65	0.90
OM digestibility	671.0	N50	89.5	86.0	80.6	91.2	86.8	86.9	0.4^{ns}	-1.3***	1.0*
	(g kg^{-1} DM)	N150	96.7	85.5	82.9	90.0	88.7	87.6	-0.8^{ns}	-1.3***	1.0*
		N450	94.0	87.0	81.6	94.5	89.3	94.8	5.9***	-1.3***	1.0*
		s.e.	1.39	1.36	1.42	1.47	0.74	0.82	1.16	0.35	0.47
Metabolisable	10.77	N50	85.2	81.0	79.4	91.0	84.2	83.1	-0.8^{ns}	-1.3***	1.0*
energy	(MJ kg^{-1} DM)	N150	92.1	80.8	81.8	90.1	86.2	84.2	-1.8*	-1.3***	1.0*
		N450	92.5	85.5	82.5	95.6	89.0	94.7	5.9***	-1.3***	1.0*
		s.e.	1.43	1.40	1.46	1.51	0.76	0.85	1.19	0.36	0.48

*** $P \leq 0.001$, ** $P \leq 0.01$, * $P \leq 0.05$, [†] $P \leq 0.1$, ns: not significant

[‡] Lp: *L. perenne*, Dg: *D. glomerata*, Tp: *T. pratense*, Tr: *T. repens*

[§] The diversity effect is calculated as the difference between the performance of the equi-proportional four-species mixture and the average of monocultures and is split into the effects of mixing grass and legume species (D_{BGL} effect), mixing the two grasses Lp and Dg (Lp·Dg effect), and mixing the two legumes Tp and Tr (Tp·Tr effect).

[¶] Standard errors (s.e.) were calculated from the weighted average of variances at each N fertilisation level.

[#] Weed biomass and NO_3 in soil solution were natural log transformed and subsequently scaled to range between 0 and 100%; thus, scaling with the maximal functional performance is not reasonably possible. See Fig. S4 for back-transformed values on the linear scale.

[‡‡]Lp·Dg and Tp·Tr effects and were omitted for N_{sym} and NO_3 in soil solution to avoid the estimation of negative monoculture values (N_{sym}) and convergence problems (NO_3).

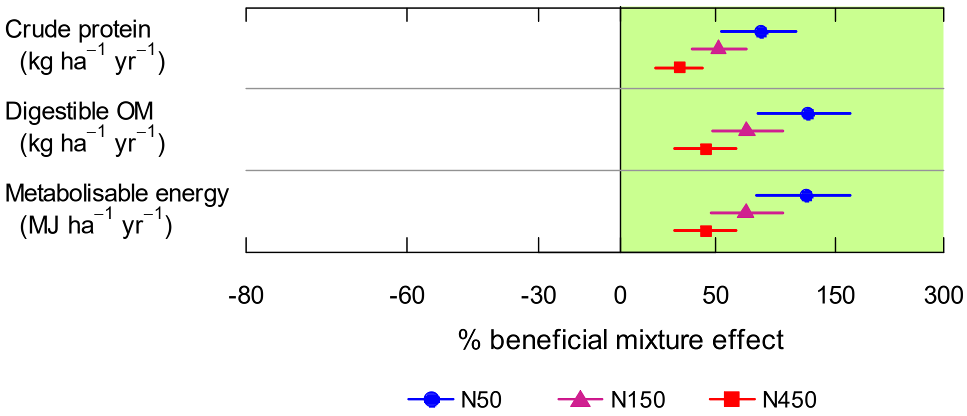

Figure S1. Percent of beneficial mixture effect (mixture performance greater than the average of monocultures) of the four-species equi-proportional mixture for the three forage quality functions scaled per hectare at three N fertilisation treatments (N50: 50 kg N ha^{-1} yr^{-1}, N150: 150 kg N ha^{-1} yr^{-1}, N450: 450 kg N ha^{-1} yr^{-1}). Data were averaged across years. OM: organic matter. Further details are explained in Fig. 2, main text.

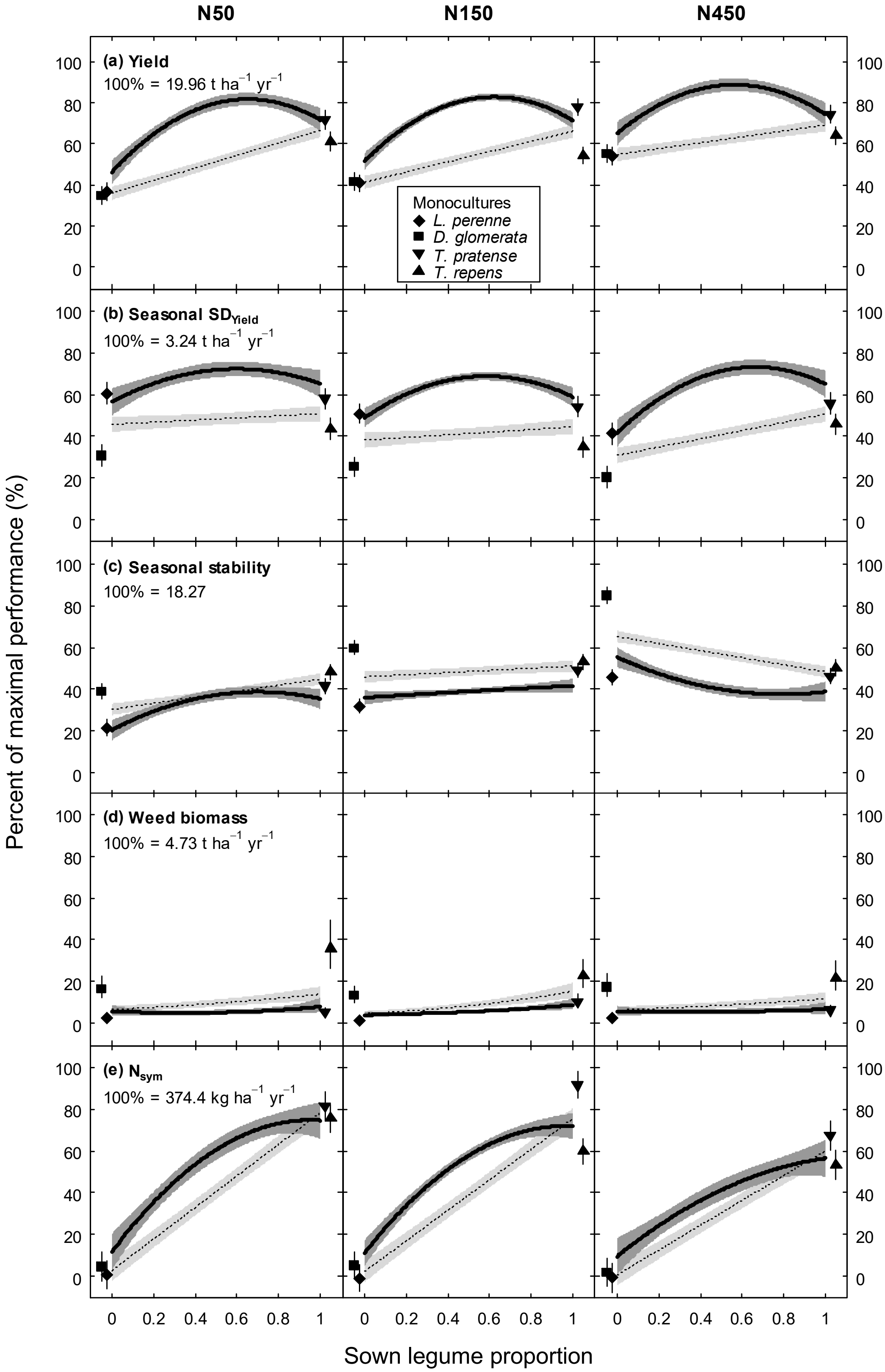

Figure S2. Predicted performance (bold lines, ±1 s.e. dark grey shaded) of seven functions in dependence on legume proportion at three N fertilisation treatments in **year 1** (scaled in % of the maximal performance per function at a single year over the three-year experiment and N fertilisation treatments; N50: 50 kg N ha^{-1} yr^{-1}, N150: 150 kg N ha^{-1} yr^{-1}, N450: 450 kg N ha^{-1} yr^{-1}).

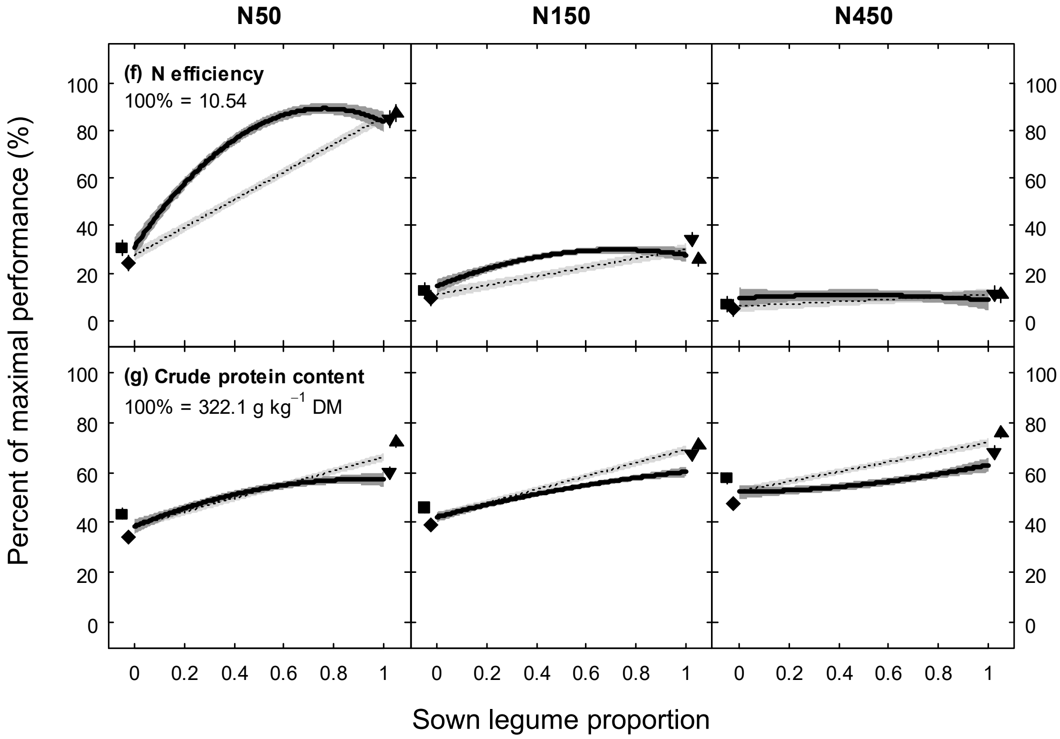

Figure S2 continued. Predictions and s.e. are based on multivariate regression analyses (Table S3) and are displayed for *mixtures that are equally composed of the two grass and the two legume species*, meaning that the predictions at a legume proportion of 0.5 are for the four-species equi-proportional mixture and at the left and right endpoints of lines for binary mixtures. Predicted performance of monocultures (±1 s.e.) is indicated by symbols. Dotted lines display the functional performance (±1 s.e. light grey shaded) that can be expected from the weighted average of monocultures in the absence of any diversity effect. Performance of weed biomass is back-transformed to linear scale.

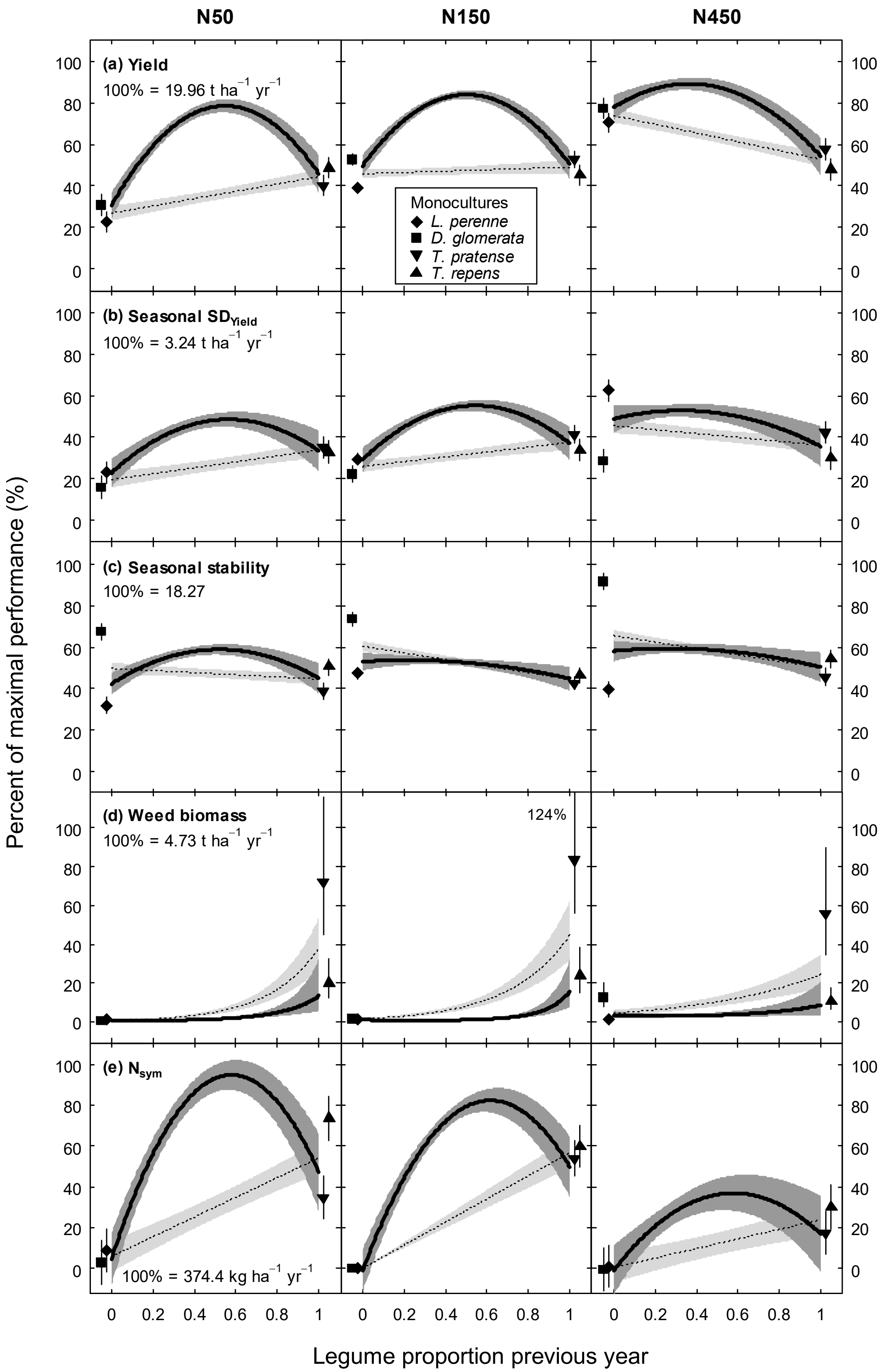

Figure S3. Predicted performance (bold lines, ±1 s.e. dark grey shaded) of ten functions in dependence on legume proportion at three N fertilisation treatments in **year 2** (scaled in % of the maximal performance per function at a single year over the three-year experiment and N fertilisation treatments; N50: 50 kg N ha^{-1} yr^{-1}, N150: 150 kg N ha^{-1} yr^{-1}, N450: 450 kg N ha^{-1} yr^{-1}).

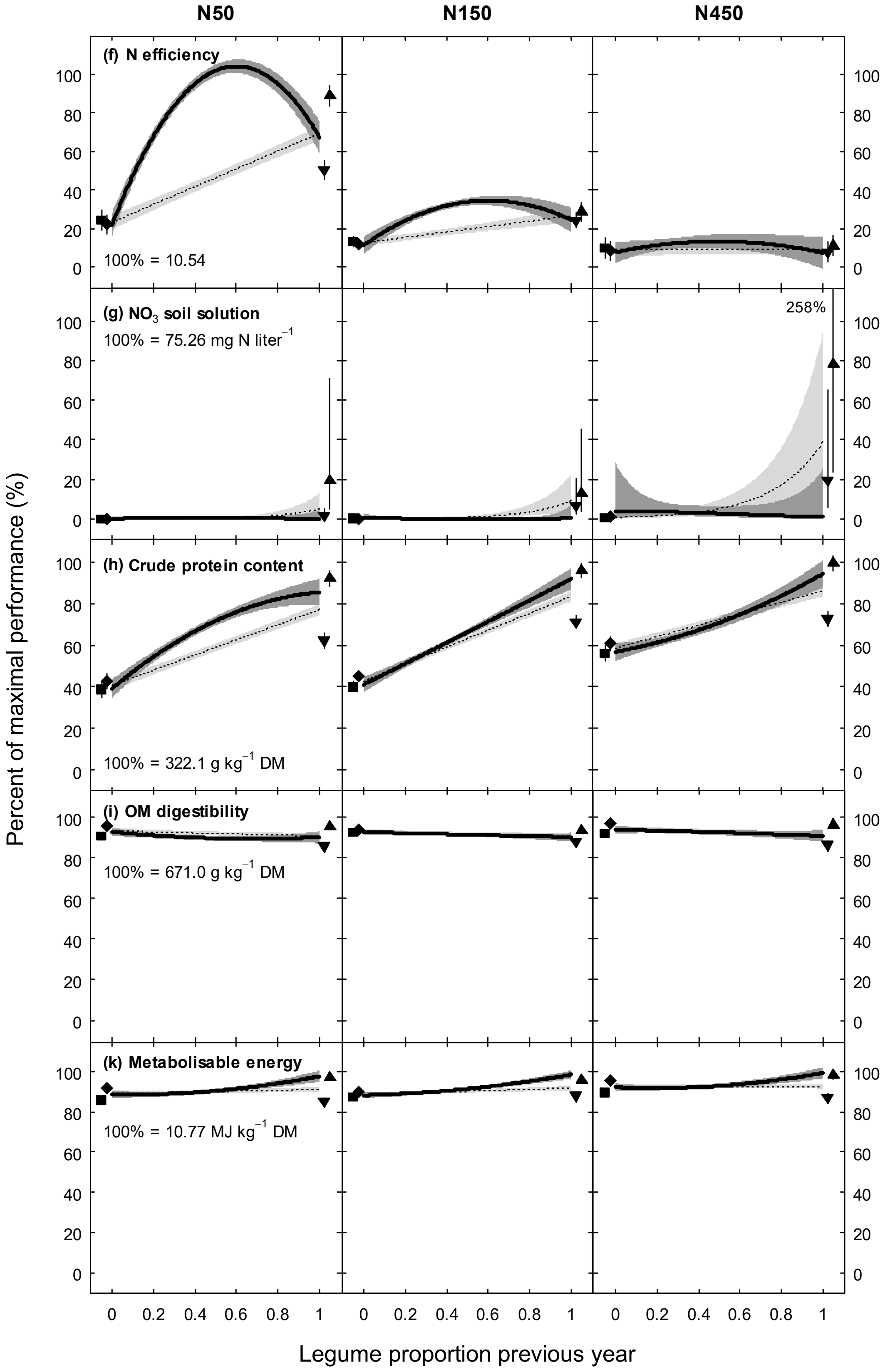

Figure S3 continued. Interpretation of lines is as explained in Fig. S2. Performance of weed biomass and NO_3 in soil solution is back-transformed to linear scale. Large upper monoculture s.e. in panels d) and g) is truncated and reported as numbers.

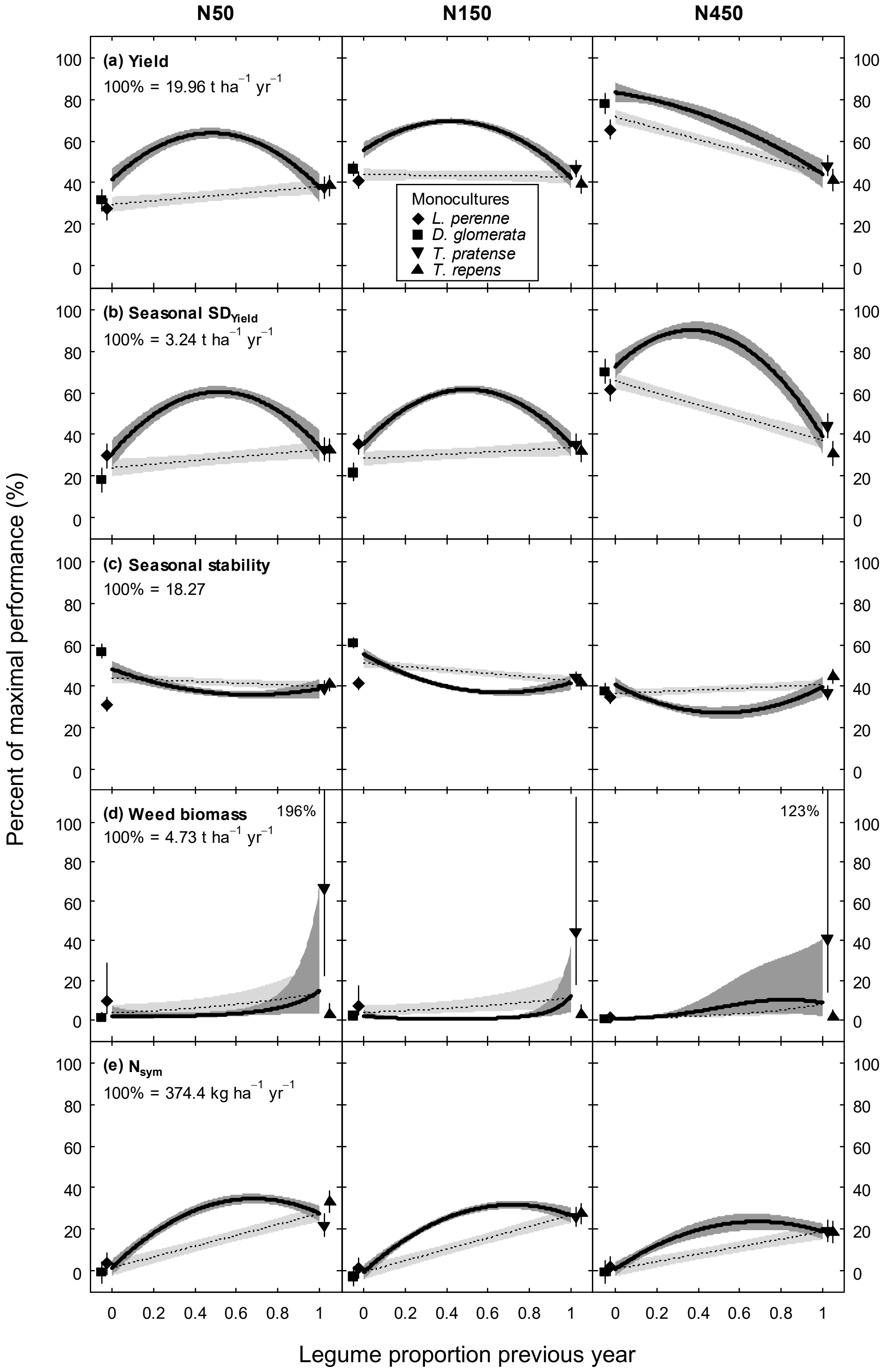

Figure S4. Predicted performance (bold lines, ±1 s.e. dark grey shaded) of ten functions in dependence on legume proportion at three N fertilisation treatments in **year 3** (scaled in % of the maximal performance per function at a single year over the three-year experiment and N fertilisation treatments; N50: 50 kg N ha^{-1} yr^{-1}, N150: 150 kg N ha^{-1} yr^{-1}, N450: 450 kg N ha^{-1} yr^{-1}).

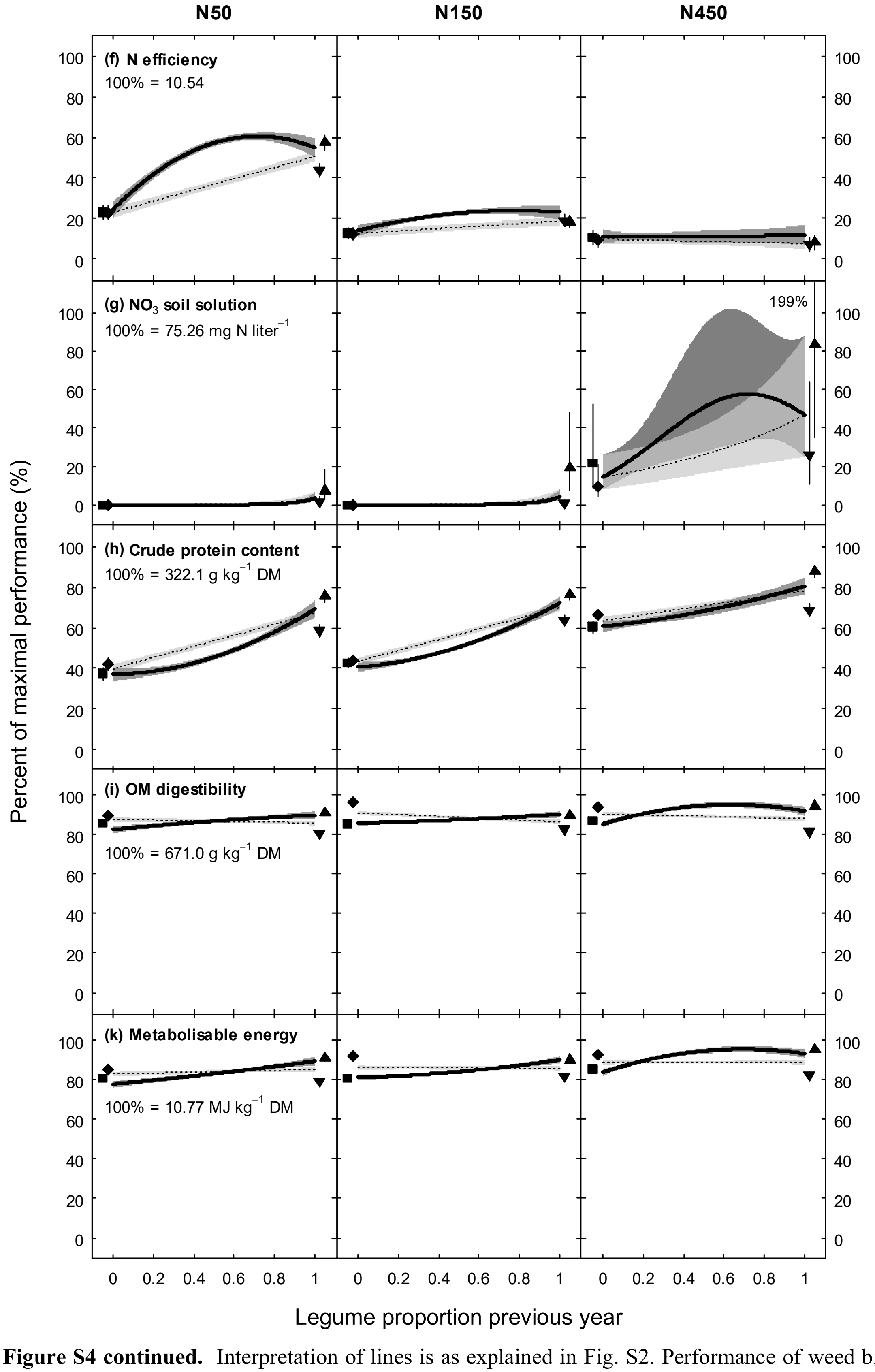

Figure S4 continued. Interpretation of lines is as explained in Fig. S2. Performance of weed biomass and NO_3 in soil solution is back-transformed to linear scale. Large upper monoculture s.e. in panels d) and g) is truncated and reported as numbers, and the intermediate gray in g) indicates the cross-section of the two s.e. bands.

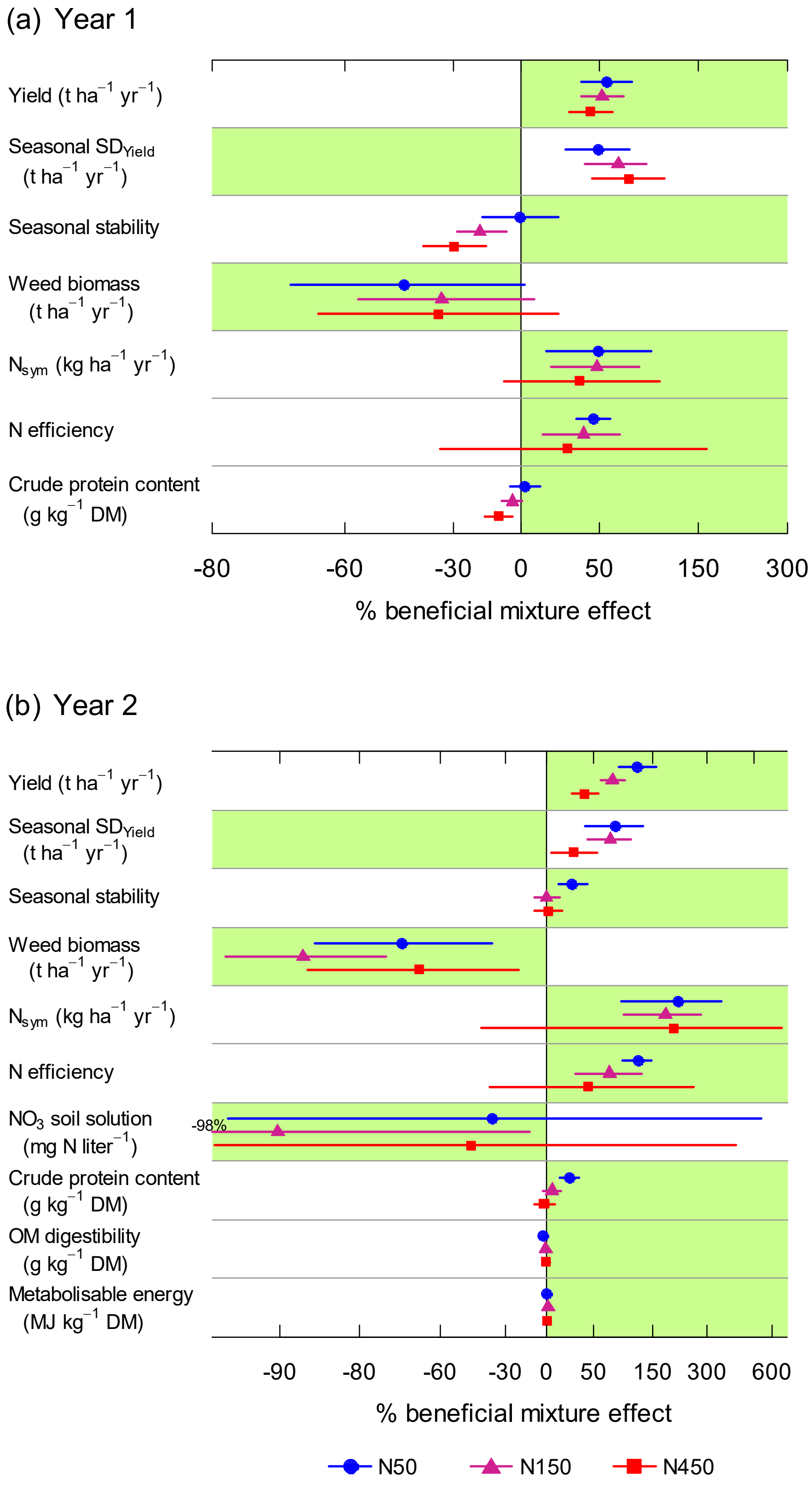

(a) Year 1
Yield (t ha−1 yr−1)
Seasonal SDYield (t ha−1 yr−1)
Seasonal stability
Weed biomass (t ha−1 yr−1)
Nsym (kg ha−1 yr−1)
N efficiency
Crude protein content (g kg−1 DM)
-80
-60
-30
0
50
150
300
% beneficial mixture effect
(b) Year 2
Yield (t ha−1 yr−1)
Seasonal SDYield (t ha−1 yr−1)
Seasonal stability
Weed biomass (t ha−1 yr−1)
Nsym (kg ha−1 yr−1)
N efficiency
NO3 soil solution (mg N liter−1)
-98%
Crude protein content (g kg−1 DM)
OM digestibility (g kg−1 DM)
Metabolisable energy (MJ kg−1 DM)
-90
-80
-60
-30
0
50
150
300
600
% beneficial mixture effect
N50
N150
N450

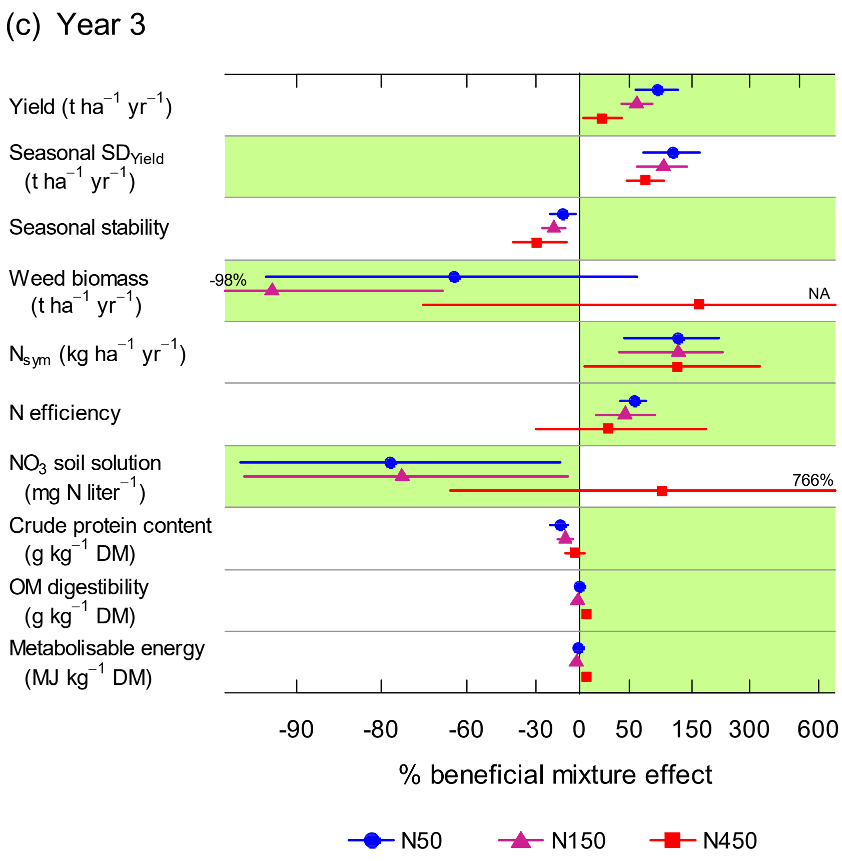

Figure S5. Percent of beneficial mixture effect (mixture performance greater than the average of monocultures) of the four-species equi-proportional mixture at three N fertilisation treatments for seven (a) and ten functions (b, c) over three experimental years (N50: 50 kg N ha^{-1} yr^{-1}, N150: 150 kg N ha^{-1} yr^{-1}, N450: 450 kg N ha^{-1} yr^{-1}). Point estimates are based on multivariate linear mixed-effects regression (Tables S3, S4, S5) and error bars represent the 95% confidence intervals (CIs). Functions whose CI does not include 0 can be considered to reveal a significant mixture effect. Weed biomass and NO_3 in soil solution were back-transformed to linear scale to calculate the beneficial mixture effect. Extreme CIs are truncated and reported as numbers, and X axes are log-scaled to equalise distances on both sides of parity. Note the different scale in (a).

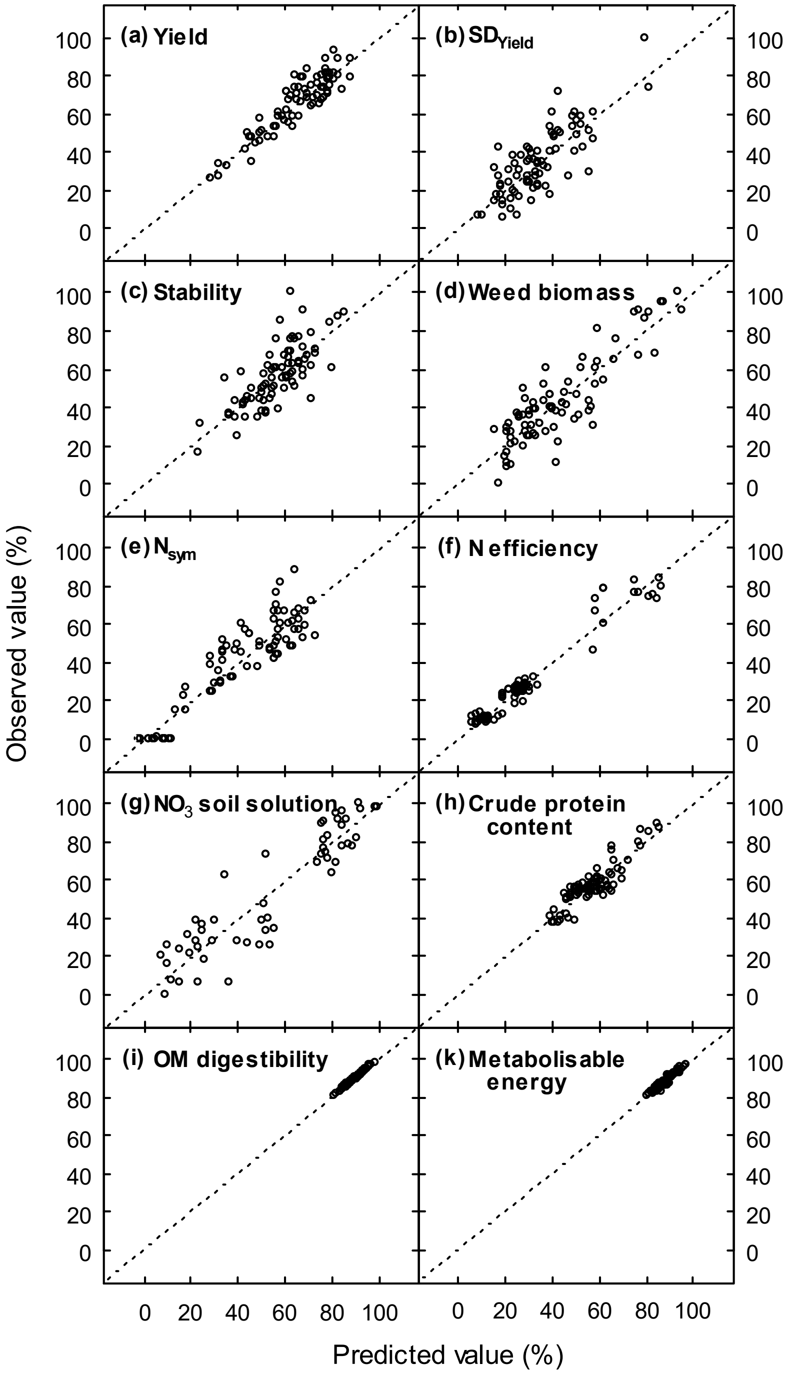

Figure S6. Observed *versus* predicted values of the ten function based on multivariate regression analyses following eqn. 3, main text. Prior to analyses, stability, weed biomass, and NO_3 in soil solution were natural log transformed to achieve a multivariate normal distribution of residuals, and all functions were standardised to range between 0 and 100%.
